# A Causal Effect of Serum 25(OH)D Level on Appendicular Muscle Mass: Evidence From NHANES Data and Mendelian Randomization Analyses

**DOI:** 10.1002/jcsm.13778

**Published:** 2025-03-31

**Authors:** Qian Ren, Jinrong Liang, Yanmei Su, Ruijing Tian, Junxian Wu, Sheng Ge, Peizhan Chen

**Affiliations:** ^1^ Department of Clinical Nutrition Shanghai Sixth People's Hospital Affiliated to Shanghai Jiao Tong University School of Medicine Shanghai China; ^2^ Department of Oncology Shanghai Sixth People's Hospital Affiliated to Shanghai Jiao Tong University School of Medicine Shanghai China; ^3^ Department of Endocrinology and Metabolism, Shanghai General Hospital Shanghai Jiao Tong University School of Medicine Shanghai China; ^4^ Clinical Research Center, Ruijin Hospital Shanghai Jiao Tong University School of Medicine Shanghai China

**Keywords:** 25‐hydroxyvitamin D, appendicular muscle mass, Mendelian randomization, NHANES, single nucleotide polymorphism

## Abstract

**Background:**

Low serum vitamin D status was reported to be associated with reduced muscle mass; however, it is inconclusive whether this relationship is causal. This study used data from the National Health and Nutrition Examination Survey (NHANES) and two‐sample Mendelian randomization (MR) analyses to ascertain the causal relationship between serum 25‐hydroxyvitamin D [25(OH)D] and appendicular muscle mass (AMM).

**Methods:**

In the NHANES 2011–2018 dataset, 11 242 participants (5588 males and 5654 females) aged 18–59 years old were included, and multivariant linear regression was performed to assess the relationship between 25(OH)D and AMM measured by dual‐energy X‐ray absorptiometry. In two‐sample MR analysis, 167 single nucleotide polymorphisms significantly associated with serum 25(OH)D at the genome‐wide association level (*p* < 5 × 10^−8^) were applied as instrumental variables (IVs) to assess vitamin D effects on AMM in the UK Biobank (417 580 Europeans) using univariable and multivariable MR (MVMR) models.

**Results:**

In the NHANES dataset, serum 25(OH)D concentrations were positively associated with AMM (*β* = 0.013, SE = 0.001, *p* < 0.001) in all participants, after adjustment for age, race, season of blood collection, education, income, body mass index and physical activity. In stratification analysis by sex, males (*β* = 0.024, SE = 0.002, *p* < 0.001) showed more pronounced positive associations than females (*β* = 0.003, SE = 0.002, *p* = 0.024). In univariable MR, genetically higher serum 25(OH)D levels were positively associated with AMM in all participants (*β* = 0.049, SE = 0.024, *p* = 0.039) and males (*β* = 0.057, SE = 0.025, *p* = 0.021), but only marginally significant in females (*β* = 0.043, SE = 0.025, *p* = 0.090) based on IVW models was noticed. No significant pleiotropy effects were detected for the IVs in the two‐sample MR investigations. In MVMR analysis, a positive causal effect of 25(OH)D on AMM was observed in the total population (*β* = 0.116, SE = 0.051, *p* = 0.022), males (*β* = 0.111, SE = 0.053, *p* = 0.036) and females (*β* = 0.124, SE = 0.054, *p* = 0.021).

**Conclusions:**

Our results suggested a positive causal effect of serum 25(OH)D concentration on AMM; however, more researches are warranted to unveil the underlying biological mechanisms and evaluate the effects of vitamin D intervention on AMM.

## 1 Introduction

The quantity and quality of skeletal muscle mass regulate whole‐body metabolic health and maintain physiological functions, such as movement, posture maintenance and thermogenesis [1]. Skeletal muscle mass diminishes continuously with age, which leads to sarcopenia in elderly people. Low skeletal muscle mass and weakness result in several physical and psychological disorders, including depression, falls, frailty, disability and even mortality [1]. Enhancement of skeletal muscle health is critical for the improvement of life quality.

Apart from the well‐established roles in maintaining bone and mineral homeostasis, growing evidence manifests that serum vitamin D levels may correlate to skeletal muscle mass [2, 3]. Observational and cohort studies have reported an association between poor vitamin D status and muscle mass loss [4, 5]. A Korean cohort study found an inverse association of 25‐hydroxyvitamin D [25(OH)D] with muscle mass both in young and elder individuals [5]. Wen et al. found participants with decreased circulating 25(OH)D (< 30 ng/mL) exhibited more serious quadriceps‐fibred cross‐sectional area loss after surgery than those with 25(OH)D ≥ 30 ng/mL [4]. Theoretically, poor vitamin D status may aggravate muscle loss through influencing the vitamin D receptor (VDR) signalling pathway, increasing oxidative stress, promoting proteolysis and cellular senescence, impairing mitochondrial function and activating the renin–angiotensin system [2, 6, 7]. Nonetheless, whether this association is causal or not is still unclear; moreover, conflicting results were observed in preceding clinical intervention studies and systematic reviews that evaluated the effects of vitamin D supplements on skeletal muscle mass [8, 9, 10].

Given the inconclusive results obtained by epidemiological studies thus far, there is an urgent need for more sophisticated studies to estimate the causal effects of serum 25(OH)D on appendicular muscle mass (AMM). The MR method, which fundamentally uses inherited genetic variants as the instrumental variables (IVs) of possible exposures to investigate their associations with the outcomes and shield against unaccounted confounding factors and reverse causality, can be utilized to infer causality [11, 12].

In the present study, we first explored the relationship between serum 25(OH)D and AMM in US adults who were recruited in the National Health and Nutrition Examination Survey (NHANES) from 2011 to 2018. We further applied the 167 independent single nucleotide polymorphisms (SNPs) that were significantly associated with serum 25(OH)D in a recent genome‐wide association meta‐analysis (GWAS) as the genetic IVs to evaluate the causal relationship between 25(OH)D and AMM [13]. We also performed a multivariable MR (MVMR) analysis to assess the confounders, including BMI, education attainment, income and physical activities, that may influence the causal associations of 25(OH)D on AMM. The results may provide evidence to support increasing the vitamin D levels for the improvement of skeletal muscle mass in the future.

## 2 Methods

### 2.1 NHANES Study Design and Data Sources

**FIGURE 1:**
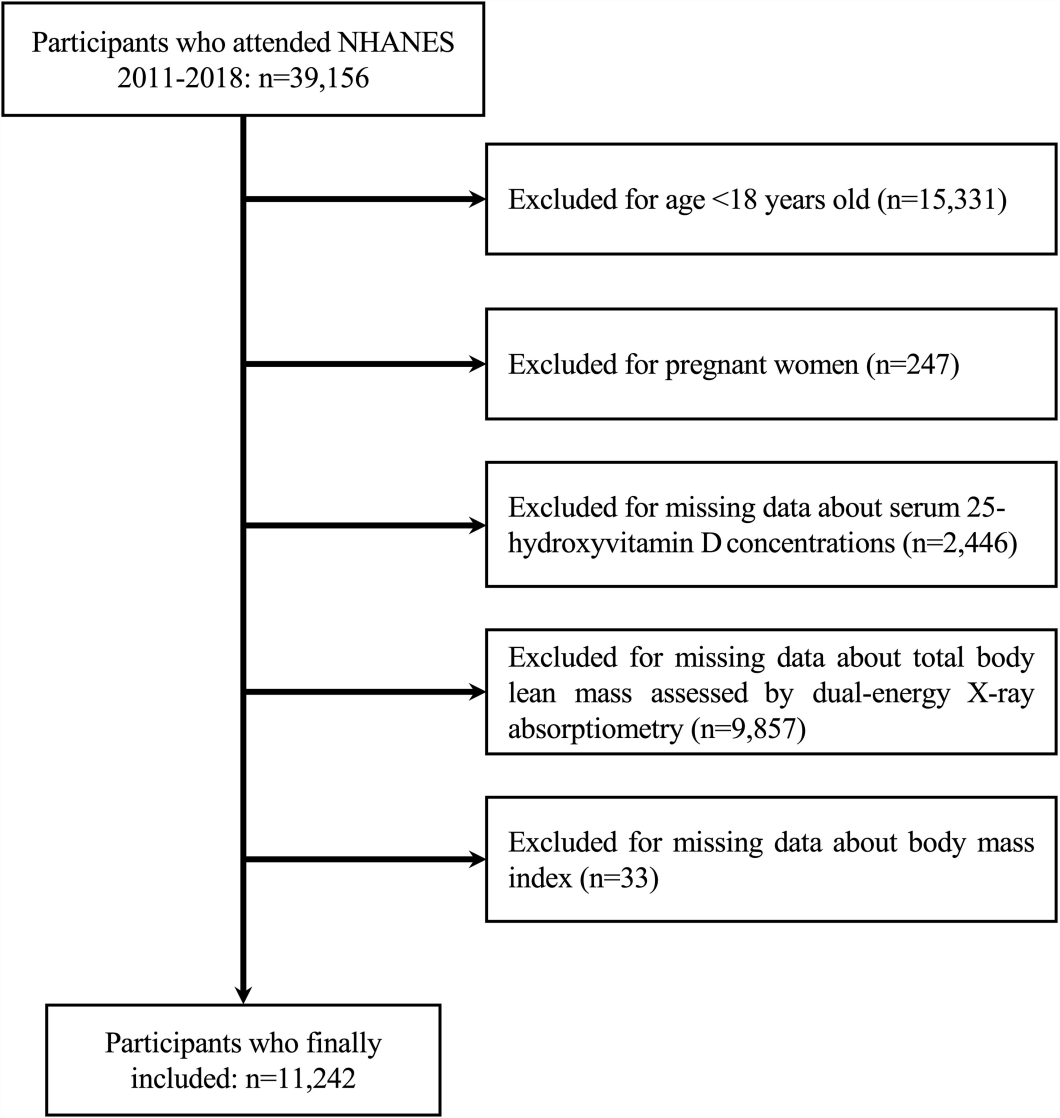
Working flowchart of participants selection in the cross‐sectional study. The current study included 39 156 participants who were recruited between 2011 and 2018. Participants who were pregnant (*n* = 247), younger than 18 years old (*n* = 15 331), lacking information on vitamin D concentration (*n* = 2446), dual‐energy X‐ray absorptiometry values (*n* = 9857) or body mass index (*n* = 33) were excluded. Finally, a total of 11 242 participants were included to evaluate the associations between vitamin D status and appendicular muscle mass.

The NHANES is a nationwide survey aimed at evaluating nutritional status and health outcomes in the United States [14]. Since 1999, the poll has been conducted in 2‐year cycles, with an annual count of about 5000 participants [14]. The current study included 39 156 participants who were recruited between 2011 and 2018. Participants who were pregnant (*n* = 247), under the age of 18 years old (*n* = 15 331), lacking information regarding serum 25(OH)D concentration (*n* = 2446), body lean mass parameters (*n* = 9857) and body mass index (BMI) (*n* = 33) were excluded. Finally, 11 242 participants were included in our NHANES dataset (Figure 1). Each participant provided written informed consent, and all procedures were approved by the institutional review board of the National Center for Health Statistics (accessible at https://www.cdc.gov/nchs/nhanes/irba98.html).

### 2.2 Measurement of AMM

The golden standard for determining body composition is dual‐energy X‐ray absorptiometry (DXA) because of its effectiveness, practicality and the low radiation dose [15]. In the 2011–2018 NHANES, whole‐body DXA was carried out on individuals aged 8–59 years, except pregnant women, those who had received a radiographic contrast test (barium) within the past 7 days and those who weighed more than 450 pounds or stood taller than 6 ft 5 in. [16]. Model Hologic Discovery DXA scanning was done using a densitometer (Hologic, Inc., Bedford, MA, USA) along with quality control evaluation [16]. The current analysis of AMM was calculated by the total lean mass in the arms and legs excluding bone mass. The AMM index (AMI) was calculated by AMM (kg)/height (m)^2^.

### 2.3 Measurement of Serum 25(OH)D Levels

Tandem mass spectrometry with high‐performance liquid chromatography was used to measure the serum 25(OH)D concentrations [16, 17]. The methodology had been previously published, and it was reliable for measuring serum 25(OH)D levels between 0 and 250 nM [18, 19]. Vitamin D status was divided into three groups according to serum 25(OH)D levels: sufficient (≥ 75.0 nM), insufficient (50.0–74.9 nM) and deficient (< 50.0 nM) groups [20].

### 2.4 Assessment of Covariates

Self‐reported questionnaires were used to gather information on age, sex, race, educational attainment, physical activity, income and smoking history. Race was categorized as Mexican American, non‐Hispanic White, other Hispanic, non‐Hispanic Black and other races [16]. Education attainments were divided into three categories, including below high school, high school or equivalent, and college or beyond [16]. Family income was divided by poverty guidelines for the survey year to calculate the poverty index ratio (PIR), which reflected household income relative to national poverty thresholds [21]. The PIR was classified as 0–1.0, 1.0–3.0 and > 3.0, with a PIR < 1.0 corresponding to a household with an income below the poverty line [17]. Smoking history was categorized as current smoker, former smoker or never smoker [16]. Throughout the blood collection and analysis process, strict protocols had been followed [17].

### 2.5 Two‐Sample MR Study Design and Data Sources

**FIGURE 2:**
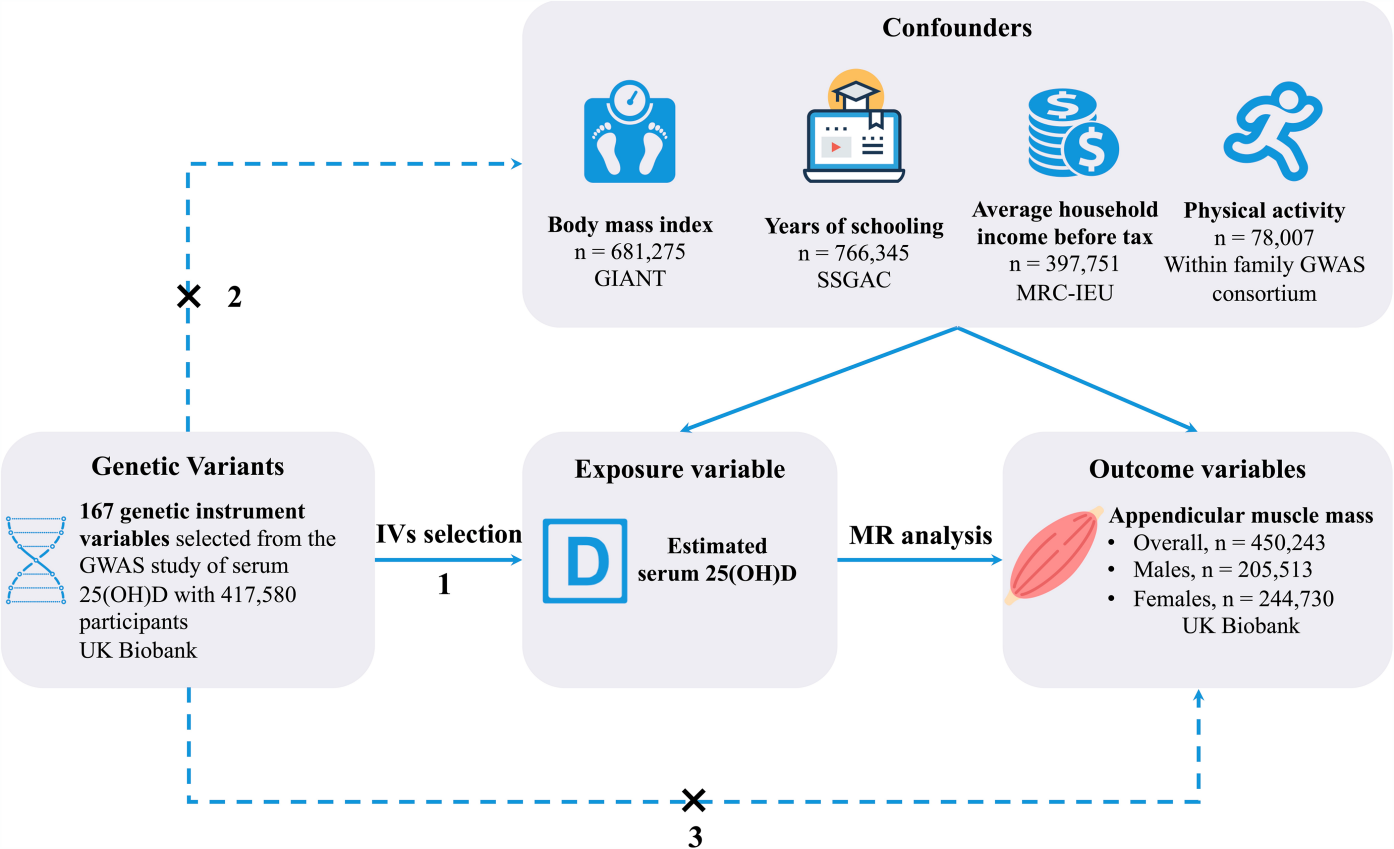
The study assumptions of the two‐sample Mendelian randomization analysis between serum 25(OH)D and appendicular muscle mass. The assumptions include: (1) the genetic instrumental variables (IVs) should exhibit a significant association with serum 25(OH)D; (2) the genetic IVs should not associate with any other potential confounding factors; and (3) the genetic IVs must only through serum 25(OH)D but not any other confounders to influence the appendicular muscle mass. The dotted lines indicate the violate of the assumptions. 25‐hydroxyvitamin D, 25(OH)D; GIANT, Genetic Investigation of Anthropometric Traits; MRC‐IEU, MRC Integrative Epidemiology Unit; SSGAC, Social Science Genetic Association Consortium.

We used the public summary‐level data from the GWASs as the basis for two‐sample MR investigations to assess the causal relationship between serum 25(OH)D and AMM. Genetic IVs originated from a GWAS conducted by Revez et al., and the summary‐level data from a GWAS research involving 417 580 people of European ancestry in the UK Biobank database was used to evaluate the causal relationship between serum 25(OH)D level and AMM in the general population, males or females [13]. The study design of the two‐sample MR analyses was provided in Figure 2. The majority of individuals in the GWAS datasets were of European ancestry, and the information on participant characteristics as well as detailed data sources for exposure, confounders and outcomes were listed in Table S1.

Because the two‐sample MR analyses were carried out using summary‐level data from publicly available GWASs, our in‐house institute ethics committee approval is not required. It was considered that each GWAS had obtained the written consent from each participant. This study complied with the reporting standards established by Strengthening the Reporting of Observational Studies in Epidemiology Using Mendelian Randomization (STROBE‐MR).

### 2.6 Selection and Validation of IVs

The MR study design stipulates that the IVs used to evaluate the causal relationship between 25(OH)D and AMM must satisfy three criteria: (1) exhibit a significant association with serum 25(OH)D; (2) not associated with any other potential confounding factors; and (3) exhibit no direct correlation between the IVs and AMM or any other mechanisms other than exposure (Figure 2). Here, possible genetic IVs were identified as SNPs from a substantial GWAS performed in UK Biobank [13]. According to the report by Revez et al., a meta‐analysis involving 417 580 individuals with European ancestry had identified a total of 18 864 distinct variants located on 143 separate loci that were significantly associated with vitamin D [13]. The base pair positions of these SNPs were determined by clumping all associated variants across a 1‐Mb window on chromosomes (linkage disequilibrium [LD] *r* ^2^ < 0.1), indicating these IVs met the Assumptions 1 and 2 of the two‐sample MR study design [13]. A total of 167 independent SNPs that reached the genome‐wide significance (*p* < 5 × 10^−8^) were recognized as genetic IVs in our two‐sample MR study (Table S2). Finally, we used the following formula to get *R* ^2^: (2 × EAF × (1 − EAF) × *β* ^2^), where *β* is the expected genetic influence on serum 25(OH)D and EAF is the effect allele frequency [22]. Using the formula *F* = (*R* ^2^ × (*n* − 1 − *k*))/((1 − *R* ^2^) × *k*) as stated, we computed the *F*‐statistic to evaluate the strength of each IVs [22]. Here, *R* ^2^ stands for the fraction of phenotype variation explained by genetic variants, *k* for the number of IVs and *n* for the sample size [22]. It was advised to adopt an *F*‐statistic > 10 in the MR study because it was regarded as a robust IV [12]. These genetic IVs had sufficient strength in the two‐sample MR studies, as *F*‐statistic values ranged from 23.985 to 1195.857 (Table S2).

### 2.7 Genetic Associations Between IVs and AMM

The TwoSampleMR package (Version 0.5.6) in R (www.r‐project.org) was used to obtain summary‐level data for associations between each genetic IV and total, male and female AMM from the UK Biobank GWAS database (https://gwas.mrcieu.ac.uk/) [23]. Summary data for the associations between serum 25(OH)D–associated genetic variants with AMM were obtained from a GWAS with 450 243 participants (including 205 513 males and 244 730 females) of European ancestry, with study IDs ‘ebi‐a‐GCST90000025’, ‘ebi‐a‐GCST90000026’ and ‘ebi‐a‐GCST90000027’ for the overall population, males and females, respectively. If an SNP for an exposed phenotype was not available in AMM summary statistics, it was replaced by another proxy SNP in high LD (*r* ^2^ > 0.80) with validated IVs as determined using the 1000 Genomes Reference Panel in individuals of European ancestry. As per the design of the two‐sample MR investigation, the IVs met the third assumption as none of them showed significant genome‐wide association with AMM (*p* < 5 × 10^−8^).

### 2.8 Genetic IVs for Confounders

To account for the confounders, including BMI, education attainment, income and physical activity, that may influence the causal associations of 25(OH)D on AMM, MVMR analysis was also performed. We extracted SNPs that were significantly associated (*p* < 5 × 10^−8^) with BMI (*n* = 681 275, ‘ieu‐b‐40’, GIANT) [24], years of schooling (*n* = 766 345, ‘ieu‐a‐1239’, SSGAC) [25], average household income before tax (*n* = 397 751, ‘ukb‐b‐7408’, MRC‐IEU) [26] and physical activity (*n* = 78 007, ‘ieu‐b‐4860’, within family GWAS consortium) through querying the IEU GWAS database (https://gwas.mrcieu.ac.uk) using the TwoSampleMR package [27]. As final IVs, we further refined the data to eliminate any SNPs in a 10 000 kb window of the genomic region that had a pairwise LD *r* ^2^ > 0.001.

### 2.9 Statistical Analyses

Continuous variables were presented as mean ± standard deviation (SD), while categorical variables were presented as numbers and their corresponding proportions. Differences between male and female groups were analysed by independent‐sample *t*‐test or *χ* ^2^ test. The relationship between vitamin D status and AMM was evaluated using multiple linear regression models, after adjusting for age, sex, race, education attainment, PIR, season of blood collection, smoking history and physical activity. As AMM existed, the difference between males and females, we also performed the multiple linear regression models stratified by sex after adjusting for the confounding variables listed above. Statistical analyses were performed using R (Version 4.1.1; www.r‐project.org) with the ‘*nlme*’ package (Version 3.1‐164).

We generated MR estimates using the traditional inverse variance weighted (IVW) method, IVW radial, fixed‐effects IVW model and the simple median approach to ascertain the causal associations between blood 25(OH)D and AMM [28]. For these genetic IVs, we used the MR‐Egger approach to assess the evidence of pleiotropic effects, which occurred when the IVs affected a condition apart from the exposure in MR investigations [29]. Significant departures from the origin of the intercept suggest the possibility of pleiotropy effects. To account for the potential confounders in assessing 25(OH)D and AMM by confounders, we also performed the MVMR analysis adjusted for BMI, education attainment, income and physical activity. The models incorporated the genome‐wide significant signals of these exposures, independent of the 25(OH)D‐related variations (*R* ^2^ LD < 0.1, based on the 1000 Genome panel reference).

We employed Cochran's Q‐statistic under the traditional IVW approach to evaluate heterogeneity between the causal estimates inferred by the IVs. To determine whether a single SNP under the traditional IVW model had an impact on the causation estimate, the leave‐one‐out sensitivity method was applied. To ascertain the validity of the hypothesis that exposure [serum 25(OH)D] has a positive association with the outcome (AMM), an MR‐Steiger directionality test was conducted. TwoSampleMR package (Version 0.5.6) of R (Version 4.1.1) was used for data acquisition and two‐sample MR analyses. All statistical tests were two‐sided and considered statistically significant when *p* < 0.05.

## 3 Results

### 3.1 Characteristics of Participants in NHANES 2011–2018 Datasets

The characteristics and muscle mass parameters of the included participants were grouped by sex (Table 1). There were 11 242 adults (5588 males and 5654 females) with a mean age of 37.73 ± 12.39 years old. Overall, poor vitamin D status was noticed in 79.60% of subjects, with 37.56% of vitamin D insufficiency and 42.04% of vitamin D deficiency. Approximately one‐fifth of the individuals were below the poverty level (with a PIR < 1.0). The sedentary time was significantly longer for females than males (*p* = 0.026). Moreover, the weight, arms, legs, trunk and total lean percentage, AMM and AMI were lower in women than in men (all *p* < 0.001); however, BMI was higher in women than in men (*p* < 0.001).

**TABLE 1:** Characteristics and muscle mass of participants from NHANES 2011–2018 (*n* = 11 242).

	Total (*n* = 11 242)	Male (*n* = 5588)	Female (*n* = 5654)	*p* value
Age (years, mean ± SD)^a^	37.73 ± 12.39	37.31 ± 12.46	38.15 ± 12.30	< 0.001
Race, *n* (%)^b^				0.001
Mexican American	1766 (15.71)	868 (15.53)	898 (15.88)	
Other Hispanic	1185 (10.54)	536 (9.59)	649 (11.48)	
Non‐Hispanic White	3866 (34.39)	1921 (34.38)	1945 (34.40)	
Non‐Hispanic Black	2304 (20.49)	1136 (20.33)	1168 (20.66)	
Other races	2121 (18.87)	1127 (20.17)	994 (17.58)	
Education level, *n* (%)^b^				< 0.001
Less than high school	2188 (19.46)	1182 (21.15)	1006 (17.79)	
High school or equivalent	2605 (23.17)	1414 (25.31)	1191 (21.06)	
College or beyond	6449 (57.37)	2992 (53.54)	3457 (61.14)	
Poverty index ratios, *n* (%)^b^				0.007
≤ 1.0	2470 (21.97)	1152 (20.62)	1318 (23.31)	
1.0–3.0	4094 (36.42)	2074 (37.12)	2020 (35.73)	
> 3.0	3757 (33.42)	1890 (33.82)	1867 (33.02)	
Missing data	921 (8.19)	472 (8.44)	449 (7.94)	
Smoking history, *n* (%)^b^				< 0.001
Current smoker	1862 (16.56)	1050 (18.79)	812 (14.36)	
Ever smoker	509 (4.53)	340 (6.09)	169 (2.99)	
Never smoker	1752 (15.58)	1053 (18.84)	699 (12.36)	
Missing data	7119 (63.33)	3145 (56.28)	3974 (70.29)	
Sedentary time (h/day, mean ± SD)^a^	6.20 ± 3.40	6.13 ± 3.38	6.27 ± 3.41	0.026
Months of blood collection, *n* (%)^b^				0.069
November–March	5508 (48.99)	2786 (49.86)	2722 (48.14)	
April–October	5734 (51.01)	2802 (50.14)	2932 (51.86)	
25(OH)D (nM, mean ± SD)^a^	56.98 ± 24.86	55.83 ± 22.43	58.11 ± 27.00	< 0.001
Sufficient, *n* (%)^b^	2294 (20.40)	1000 (17.90)	1294 (22.89)	< 0.001
Insufficient, *n* (%)^b^	4222 (37.56)	2263 (40.50)	1959 (34.65)	
Deficient, *n* (%)^b^	4726 (42.04)	2325 (41.60)	2401 (42.47)	
Weight (kg, mean ± SD)^a^	80.63 ± 21.00	85.72 ± 19.99	75.59 ± 20.76	< 0.001
BMI (kg/m^2^, mean ± SD)	28.64 ± 6.85	28.22 ± 6.07	29.06 ± 7.52	< 0.001
Arms LM percentage (%, mean ± SD)^a^	7.66 ± 1.93	9.21 ± 1.33	6.13 ± 0.97	< 0.001
Legs LM percentage (%, mean ± SD)^a^	20.59 ± 3.38	22.66 ± 2.82	18.54 ± 2.53	< 0.001
Trunk LM percentage (%, mean ± SD)^a^	30.75 ± 7.48	33.58 ± 5.86	27.95 ± 7.84	< 0.001
Total LM percentage (%, mean ± SD)^a^	64.56 ± 8.38	70.54 ± 5.90	58.70 ± 5.95	< 0.001
AMM (kg, mean ± SD)^a^	22.57 ± 6.48	26.89 ± 5.26	18.31 ± 4.41	< 0.001
AMI (kg/m^2^, mean ± SD)^a^	7.93 ± 1.77	8.85 ± 1.52	7.02 ± 1.51	< 0.001

Abbreviations: 25(OH)D, 25‐hydroxyvitamin D; AMI, appendicular muscle mass index; BMI, body mass index; LM, lean mass; SD, standard deviation.

^a^ Comparisons between two groups were performed by independent‐sample *t*‐test.

^b^ Comparisons between two groups were performed by *χ* ^2^ test.

### 3.2 Associations Between Vitamin D Status and AMM in NHANES Data

The serum 25(OH)D concentrations were positively associated with AMM (*β* = 0.013, SE = 0.001, *p* < 0.001; Table 2) in all participants, after adjustment for age, race, season of blood collection, education, PIR, BMI and physical activity. In stratified studies by sex, positive associations were observed in males (*β* = 0.024, SE = 0.002, *p* < 0.001; Table 2) as well as in females (*β* = 0.003, SE = 0.002, *p* = 0.024; Table 2). Moreover, a positive association between AMM and BMI in the total population (*β* = 0.510, SE = 0.005, *p* < 0.001), males (*β* = 0.617, SE = 0.008, *p* < 0.001) and females (*β* = 0.441, SE = 0.005, *p* < 0.001) and a negative association between AMM and sedentary times in the total population (*β* = −0.023, SE = 0.009, *p* = 0.013) and males (*β* = −0.032, SE = 0.015, *p* = 0.031) were noticed, respectively (Table 2).

**TABLE 2:** Multivariate linear regression analysis of the associations between vitamin D status and appendicular lean mass in male (*n* = 5588) and female (*n* = 5654) participants from NHANES 2011–2018.

Appendicular lean mass	Total (*n* = 11 242)		Male (*n* = 5588)		Female (*n* = 5654)	
	*β* (SE)	*p*‐value	*β* (SE)	*p*‐value	*β* (SE)	*p‐*value
25‐Hydroxyvitamin D, nM	0.013 (0.001)	< 0.001	0.024 (0.002)	< 0.001	0.003 (0.002)	0.024
Age, years	−0.063 (0.003)	< 0.001	−0.068 (0.004)	< 0.001	−0.057 (0.003)	< 0.001
Sex (female vs. male)	−9.052 (0.061)	< 0.001	—		—	
Race						
Mexican American	Reference		Reference		Reference	
Other Hispanic	0.634 (0.125)	< 0.001	0.561 (0.200)	0.005	0.660 (0.144)	< 0.001
Non‐Hispanic White	1.358 (0.101)	< 0.001	1.271 (0.157)	< 0.001	1.529 (0.121)	< 0.001
Non‐Hispanic Black	3.647 (0.108)	< 0.001	4.325 (0.169)	< 0.001	3.217 (0.128)	< 0.001
Other races	0.320 (0.112)	0.004	0.297 (0.173)	0.087	0.450 (0.135)	< 0.001
Season (spring and summer vs. autumn and winter)	0.057 (0.062)	0.359	0.124 (0.097)	0.203	−0.017 (0.074)	0.815
Education						
Below high school	Reference		Reference		Reference	
High school or equivalent	0.167 (0.097)	< 0.001	0.034 (0.145)	0.814	0.256 (0.121)	0.035
College or above	0.759 (0.090)	< 0.001	0.769 (0.138)	< 0.001	0.730 (0.110)	< 0.001
Poverty index ratios						
≤ 1.0	Reference		Reference		Reference	
1.0–3.0	0.307 (0.080)	< 0.001	0.347 (0.126)	0.006	0.206 (0.094)	0.028
>3.0	0.667 (0.089)	< 0.001	0.742 (0.139)	< 0.001	0.437 (0.105)	< 0.001
Body mass index, kg/m^2^	0.510 (0.005)	< 0.001	0.617 (0.008)	< 0.001	0.441 (0.005)	< 0.001
Sedentary time, hour/day	−0.023 (0.009)	0.013	−0.032 (0.015)	0.031	−0.011 (0.011)	0.307

*Note:* The multivariate linear regression model was adjusted for age, race, the season of blood collection, education, poverty index ratio, body mass index and sedentary time.

Abbreviation: SE, standard error.

We also examined the associations between vitamin D status and AMI and found serum 25(OH)D concentrations were positively associated with AMI in the total population (*β* = 0.003, SE = 3.686 × 10^−4^, *p* < 0.001), males (*β* = 0.006, SE = 0.001, *p* < 0.001) and females (*β* = 0.001, SE = 4.409 × 10^−4^, *p* = 0.017; Table S3).

### 3.3 Causal Inference by the Two‐Sample Univariable and MVMR Analysis

Using two‐sample MR analysis, we assessed the causal association between serum 25(OH)D concentrations and AMM as determined by the DXA. Genetically higher serum 25(OH)D levels had a positive effect on AMM in all participants under the conventional IVW model (*β* = 0.049, SE = 0.024, *p* = 0.039). In the stratification analysis, the causal association between serum 25(OH)D and AMM was more pronounced in males (*β* = 0.057, SE = 0.025, *p* = 0.021) than in females (*β* = 0.043, SE = 0.025, *p* = 0.090) under the IVW model (Table 3). Similar results were observed in IVW radial and simple median models (Table 3). In the IVW fixed effects model, the causal association between serum 25(OH)D and AMM was also observed in females (*β* = 0.043, SE = 0.009, *p* < 0.001) and males (*β* = 0.057, SE = 0.010, *p* < 0.001) (Table 3). According to the MR‐Egger test, no significant pleiotropy effects were observed for the IVs (*p* = 0.333, *p* = 0.261 and *p* = 0.455 in the total population, males and females, respectively) in the two‐sample MR studies (Table 3), and no individual IVs significantly affected the overall estimates both in the total and stratification analyses as suggested by leave‐one‐out analyses (Tables S4–S6).

**TABLE 3:** Estimates of the two‐sample MR analyses for causal associations between 25(OH)D and appendicular lean mass using summary GWAS data in UK Biobank (*n* = 417 580).

Outcomes	SNPs	Participants	MR estimates								Pleiotropy effects	
Appendicular lean mass			IVW		IVW radial		Simple median		IVW (fixed effects)		MR‐Egger regression intercept	
			*β* (SE)	*p*‐value	*β* (SE)	*p*‐value	*β* (SE)	*p*‐value	*β* (SE)	*p*‐value	Intercept (SE)	*p*‐value
Total population	165^a^	450 243	0.049 (0.024)	0.039	0.049 (0.024)	0.039	0.038 (0.016)	0.020	0.049 (0.007)	< 0.001	−0.035 (0.036)	0.333
Male	166^b^	205 513	0.057 (0.025)	0.021	0.057 (0.025)	0.021	0.049 (0.023)	0.036	0.057 (0.010)	< 0.001	−0.043 (0.038)	0.261
Female	166^b^	244 730	0.043 (0.025)	0.090	0.043 (0.025)	0.090	0.020 (0.020)	0.331	0.043 (0.009)	< 0.001	−0.029 (0.039)	0.455

Abbreviations: 25(OH)D, 25‐hydroxyvitamin D; IVW, inverse variance weighted; MR, Mendelian randomization; SE, standard error; SNP, single nucleotide polymorphism.

^a^ rs77037130 was excluded because of missing data of association with AMM in UK Biobank for all participants and rs1011896 was excluded because of palindromic with intermediate allele frequency.

^b^ rs1011896 was excluded because of palindromic with intermediate allele frequency.

Further, the results from the MVMR analyses also suggested that serum 25(OH)D was significantly associated with AMM in the total population (*β* = 0.116, SE = 0.051, *p* = 0.022), males (*β* = 0.111, SE = 0.053, *p* = 0.036) and females (*β* = 0.124, SE = 0.054, *p* = 0.021) after adjustment of BMI, education attainment, household income and physical activities (Table 4).

**TABLE 4:** Multivariate Mendelian randomization analysis of the causal associations between vitamin D status and appendicular lean mass.

	Total population (*n* = 450 243)		Male (*n* = 205 513)		Female (*n* = 244 730)	
	*β* (SE)	*p*‐value	*β* (SE)	*p*‐value	*β* (SE)	*p*‐value
25‐Hydroxyvitamin D (ebi‐a‐GCST90000617)	0.116 (0.051)	0.022	0.111 (0.053)	0.036	0.124 (0.054)	0.021
Body mass index (ieu‐b‐40)	0.255 (0.086)	0.003	0.263 (0.089)	0.003	0.247 (0.091)	0.006
Education attainment (ieu‐a‐1239)	0.247 (0.025)	< 0.001	0.324 (0.026)	< 0.001	0.182 (0.026)	< 0.001
Household income (ukb‐b‐7408)	−0.082 (0.088)	0.539	−0.057 (0.138)	0.682	−0.107 (0.140)	0.446
Physical activity (ieu‐b‐4860)	0.233 (0.100)	0.019	0.183 (0.103)	0.076	0.272 (0.105)	0.010

## 4 Discussion

AMM measured by DXA was applied to assess the skeletal muscle mass, offering an easy‐to‐use reference for clinical and research applications [15, 30, 31]. AMM has been widely used to evaluate sarcopenia [32]. AMM, rather than trunk muscle mass, exhibited stronger correlations with swallowing function [33]. Higher predicted AMM was also associated with a reduced risk of cardiovascular diseases [34]. Wen et al. enrolled young, physically active participants with recent tears in the anterior cruciate ligament and found that reconstruction surgery led to the reduction of 25(OH)D levels and an increment of VDR and vitamin D–activating enzyme expression in skeletal muscle post‐operation [4]. Moreover, participants with poor vitamin D status [25(OH)D < 30 ng/mL] displayed more serious quadriceps fibre cross‐sectional area loss after surgery than those with better vitamin D status [25(OH)D ≥ 30 ng/mL] [4]. In the current study, we found that the serum vitamin D status was positively associated with AMM in the NHANES 2011–2018 dataset, which is consistent with previous observational studies [5]; however, whether there is a causal relationship between vitamin D and AMM and whether vitamin D intervention could improve AMM still needs to be addressed.

Several intervention studies have assessed the impacts of vitamin D supplementation on AMM. In the PROVIDE study, elderly sarcopenic individuals with mobility limitations were randomly assigned to two groups. The experimental group (*n* = 184) received a 20.7 g supplement containing 800 IU of vitamin D and leucine‐enriched whey protein per serving, whereas the control group (*n* = 196) received a 31.4 g isocaloric product without vitamin D per serving. The 13‐week intervention was conducted without additional physical exercise. The study found that the experimental group had a significantly greater increase in AMM, as assessed by DXA, compared with the control group [35]. After performing a post‐hoc analysis of the PROVIDE trial, it was found that participants with higher baseline levels of 25(OH)D and dietary protein intake experienced greater gains in AMM, AMI and relative AMM (AMM/body weight × 100%), irrespective of other determinants [36]. Similarly, in a 12‐week intervention study by Rondanelli et al., 127 elderly patients with sarcopenia (aged 81 ± 6 years) were randomly assigned to the experimental group (*n* = 64; 20 g of whey protein, 2.8 g of leucine and 800 IU of vitamin D orally once daily at mealtime) or the control group (*n* = 63; an isocaloric product) [37]. The study found significant increases in AMM and AMI in the experimental group compared with the control group [38]. In a 6‐month randomized, controlled, double‐blind trial, 115 elderly pre‐sarcopenic subjects (aged 73.31 ± 2.05 years) with 25(OH)D < 20 ng/mL were randomized into a vitamin D intervention group (10 000 IU vitamin D_3_ three times per week) or a placebo control group [37]. The vitamin D‐supplemented group showed substantially greater mean changes in AMM and handgrip strength from baseline up to 6 months compared with the placebo group. A strong positive association between the rise in plasma 25(OH)D concentration and AMM gain was also observed during the course of the 6‐month treatment period [37]. However, Prokopidis et al. compared the effects of vitamin D supplementation (1000 IU/day to 50 000 IU/month, 9–12 months) with placebo on AMM in adults more than 50 years old and found vitamin D supplementation conferred no effect on AMI [10]. Another study reported by LeBoff et al. had reviewed the literature, summarized the effects of a high dose of 2000 IU vitamin D daily on skeletal muscle outcomes in males (≥ 50 years) and females (≥ 55 years) in a 5.3‐year US VITamin D and OmegA‐3 TriaL (VITAL) trial (*n* = 25 871) and males and females (≥ 70 years) in a 3‐year European DO‐HEALTH trial (*n* = 2157), and there was no discernible improvement in muscular function after 2000 IU/day of additional vitamin D [8]. The differences in the participants baseline characteristics (such as age, race and gender), baseline vitamin D levels, physical condition, length of intervention, intervention agent (vitamin D combination or supplement alone), length of follow‐up and techniques of skeletal muscle mass assessment might underlie the inconsistent results of these population‐based studies.

In light of the mixed findings from current epidemiological research, there is a demand for more well‐designed studies to accurately evaluate the causal relationship between vitamin D and AMM. MR, a novel application of IV analysis, is gaining traction for identifying potential causal relationships by leveraging existing genetic association study data [39]. Similar to randomized controlled trial (RCT) studies, MR analyses could mitigate the risks of bias generated by confounding, measurement error and reverse causality, reduce costs and time, and may address the issues that RCT studies are unable to ask [11]. The current study employed a two‐sample MR method to provide a more reliable estimate of the causal impact of serum 25(OH)D on AMM. We noticed a significant causal effect of serum 25(OH)D on AMM in males and a marginal causal effect in females. In the MVMR analyses, the positive causal effect was observed in the total population and both genders after adjustment of BMI, education attainment, household income and physical activities. The consistent results from the cross‐sectional observation studies and two‐sample MR studies provide robust evidence for the causality between serum 25(OH)D and AMM, which can guide the clinical intervention of vitamin D in improving AMM in the general population.

There are still some limitations that should be acknowledged. First, the body composition was examined by the gold standard DXA in the current study. Compared with bioelectrical impedance analysis, significant inter‐ and intra‐individual variability in lean mass resulting from ageing and illness status can be avoided using DXA examinations. However, the DXA screening was only performed in adults aged 8–59 years in NHANES 2011–2018; therefore, we only included adults aged 18–59 years in our study. Whether the circulating 25(OH)D is associated with AMM in elderly populations is still unknown. Second, the MR study relies on aggregated GWAS data from individuals of European descent, so whether these results are consistent in older individuals or other ethnic populations requires further analysis. Third, the genetic IVs used in MR analysis are usually weak instruments, and only a small percentage of exposure variance was detected by the genetic IVs. For the causal connection between 25(OH)D concentrations and AMM in females, a marginal significance under the conventional IVW model in univariable MR analysis but an obvious significance in MVMR was observed; it may be due to methodological differences, such as bias caused by confounding between an exposure, outcome, and mediator and measurement inaccuracy [11]. Last but not least, the results could be impacted by additional confounding variables, including exposure duration and demographic stratification.

In conclusion, our results indicated a potentially causal, positive relationship between serum 25(OH)D levels and AMM. This revealed that maintaining sufficient vitamin D status may reduce the risks of AMM loss in the general population. Nevertheless, further prospective cohort or intervention trials are required to determine whether vitamin D supplementation can improve AMM, and the underlying biological mechanisms also require further investigation.

## Conflicts of Interest

The authors declare no conflicts of interest.

## Supporting information


**Table S1.** Datasets used in the two‐sample Mendelian randomization study.

**Table S2.** Summary information on serum 25‐hydroxyvitamin D associated SNPs used as genetic instrumental variables for the two‐sample Mendelian randomization analyses.

**Table S3.** Multivariate linear regression analysis of the associations between vitamin D status and appendicular lean mass index in male (*n* = 5588) and female (*n* = 5654) participants from NHANES 2011–2018.

**Table S4.** The pleiotropy effects for the genetic instrumental variables in the two‐sample Mendelian randomization studies to evaluate whether the causality estimate in the total population was affected by a single SNP using leave‐one‐out methods based on conventional inverse variance weighted model.

**Table S5.** The pleiotropy effects for the genetic instrumental variables in the two‐sample Mendelian randomization studies to evaluate whether the causality estimate in males was affected by a single SNP using leave‐one‐out methods based on conventional inverse variance weighted model.

**Table S6.** The pleiotropy effects for the genetic instrumental variables in the two‐sample Mendelian randomization studies to evaluate whether the causality estimate in females was affected by a single SNP using leave‐one‐out methods based on conventional inverse variance weighted model.
