# Supplementary material for: A Causal Effect of Serum 25(OH)D Level on Appendicular Muscle Mass: Evidence From NHANES Data and Mendelian Randomization Analyses
Source: J Cachexia Sarcopenia Muscle. 2025 Mar 31;16(2):e13778. doi: 10.1002/jcsm.13778 (PMC11955837; doi:10.1002/jcsm.13778)
Supplement: Supplementary file 1 — Table S1. Datasets used in the two‐sample Mendelian randomization study. [file JCSM-16-e13778-s003.docx]

**Supplementary Table 1.** Datasets used in the two-sample Mendelian Randomization study.

| Exposure/Outcome | Consortium | Participants and Sample size | IEU GWAS database ID |
| --- | --- | --- | --- |
| Serum 25-Hydroxyvitamin D levels | UK Biobank | a genome-wide association study of 25 hydroxyvitamin D concentration in 417,580 Europeans | ebi-a-GCST90000617 |
| Appendicular muscle mass | UK Biobank | a GWAS study with 450,243 population including 205,513 males and 244,730 females of European ancestry | ebi-a-GCST90000025 (total population);  ebi-a-GCST90000026 (males);  ebi-a-GCST90000025 (females) |
| Body mass index | Genetic Investigation of Anthropometric Traits (GIANT) | 681,275 European-descent individuals | ieu-b-40 |
| Education attainment (years of schooling) | Social Science Genetic Association Consortium (SSGAC) | 766,345 European-descent individuals | ieu-a-1239 |
| Household income (before tax) | MRC-IEU UK Biobank | European ancestry with 397,751 response individuals | ukb-b-7408 |
| Physical activity | Within family GWAS consortium | 78,007 individuals of European ancestry | ieu-b-4860 |
