# Supplementary material for: A Causal Effect of Serum 25(OH)D Level on Appendicular Muscle Mass: Evidence From NHANES Data and Mendelian Randomization Analyses
Source: J Cachexia Sarcopenia Muscle. 2025 Mar 31;16(2):e13778. doi: 10.1002/jcsm.13778 (PMC11955837; doi:10.1002/jcsm.13778)
Supplement: Supplementary file 2 — Table S2. Summary information on serum 25‐hydroxyvitamin D associated SNPs used as genetic instrumental variables for the two‐sample Mendelian randomization analyses. [file JCSM-16-e13778-s005.docx]

**Supplementary Table 2**. Summary information on serum 25-hydroxyvitamin D associated SNPs used as genetic instrumental variables for the two-sample Mendelian Randomization analyses.

| **SNP** | **Chr** | **Position** | **Effect allele** | **Other allele** | **EAF** | **UK Biobank (n = 417,580)** | | | | |
| --- | --- | --- | --- | --- | --- | --- | --- | --- | --- | --- |
|  |  |  |  |  |  | **β** | **SE** | ***P*-value** | ***R^2^*** | **F-statistic** |
| rs10908465 | 1 | 155389688 | T | C | 0.2673 | 0.0157 | 0.0022 | 1.433×10^-12^ | 9.674×10^-5^ | 40.400 |
| rs11204743 | 1 | 150890406 | T | A | 0.3320 | -0.0147 | 0.0021 | 2.143×10^-12^ | 9.583×10^-5^ | 40.019 |
| rs11264223 | 1 | 154565519 | A | G | 0.4900 | -0.0114 | 0.0020 | 6.626×10^-9^ | 6.497×10^-5^ | 27.133 |
| rs11591147 | 1 | 55505647 | T | G | 0.0180 | 0.0448 | 0.0074 | 1.304×10^-9^ | 7.098×10^-5^ | 29.643 |
| rs12123821 | 1 | 152179152 | T | C | 0.0475 | 0.0771 | 0.0046 | 9.583×10^-63^ | 5.388×10^-4^ | 225.120 |
| rs140371183 | 1 | 152098428 | G | A | 0.0322 | 0.0830 | 0.0056 | 4.056×10^-50^ | 4.294×10^-4^ | 179.403 |
| rs2131925 | 1 | 63025942 | T | G | 0.6436 | -0.0211 | 0.0021 | 9.497×10^-25^ | 2.048×10^-4^ | 85.517 |
| rs35285316 | 1 | 34691983 | T | C | 0.1699 | 0.0143 | 0.0026 | 4.842×10^-8^ | 5.744×10^-5^ | 23.985 |
| rs35408430 | 1 | 17560195 | T | C | 0.3422 | -0.0208 | 0.0021 | 1.196×10^-23^ | 1.942×10^-4^ | 81.095 |
| rs512083 | 1 | 46027355 | C | T | 0.4608 | 0.0121 | 0.0020 | 1.042×10^-9^ | 7.254×10^-5^ | 30.294 |
| rs55707527 | 1 | 41769113 | T | G | 0.2199 | 0.0152 | 0.0024 | 1.933×10^-10^ | 7.903×10^-5^ | 33.004 |
| rs6671730 | 1 | 2339139 | A | G | 0.4343 | -0.0127 | 0.0020 | 1.735×10^-10^ | 7.879×10^-5^ | 32.904 |
| rs6672758 | 1 | 230303512 | T | C | 0.8009 | 0.0146 | 0.0025 | 3.421×10^-9^ | 6.827×10^-5^ | 28.511 |
| rs7367758 | 1 | 155087083 | T | C | 0.1513 | -0.0172 | 0.0028 | 4.315×10^-10^ | 7.610×10^-5^ | 31.781 |
| rs7528419 | 1 | 109817192 | G | A | 0.2247 | 0.0199 | 0.0024 | 3.350×10^-17^ | 1.375×10^-4^ | 57.427 |
| rs78886843 | 1 | 152412328 | T | C | 0.0720 | -0.0234 | 0.0038 | 7.079×10^-10^ | 7.334×10^-5^ | 30.628 |
| rs867772 | 1 | 220972343 | G | A | 0.6845 | -0.0147 | 0.0021 | 4.282×10^-12^ | 9.341×10^-5^ | 39.008 |
| rs1047891 | 2 | 211540507 | A | C | 0.3158 | -0.0121 | 0.0021 | 9.713×10^-9^ | 6.342×10^-5^ | 26.484 |
| rs11127186 | 2 | 28881407 | C | T | 0.4958 | 0.0111 | 0.0020 | 3.201×10^-8^ | 6.157×10^-5^ | 25.713 |
| rs1260326 | 2 | 27730940 | C | T | 0.6066 | 0.0206 | 0.0020 | 1.174×10^-24^ | 2.025×10^-4^ | 84.582 |
| rs2012736 | 2 | 234622379 | A | C | 0.0808 | -0.0455 | 0.0036 | 2.579×10^-36^ | 3.078×10^-4^ | 128.560 |
| rs2710647 | 2 | 63213970 | C | T | 0.5513 | -0.0114 | 0.0020 | 7.871×10^-9^ | 6.459×10^-5^ | 26.972 |
| rs541041 | 2 | 21294975 | A | G | 0.8192 | -0.0152 | 0.0026 | 2.427×10^-9^ | 6.866×10^-5^ | 28.673 |
| rs58387006 | 2 | 32579999 | C | A | 0.2218 | -0.0134 | 0.0024 | 1.356×10^-8^ | 6.238×10^-5^ | 26.051 |
| rs6724965 | 2 | 101440151 | G | A | 0.1715 | -0.0164 | 0.0026 | 2.804×10^-10^ | 7.671×10^-5^ | 32.036 |
| rs7569755 | 2 | 118648261 | A | G | 0.2906 | 0.0136 | 0.0022 | 4.674×10^-10^ | 7.622×10^-5^ | 31.831 |
| rs7604788 | 2 | 21190024 | T | C | 0.0334 | 0.0352 | 0.0055 | 1.303×10^-10^ | 8.010×10^-5^ | 33.451 |
| rs2352974 | 3 | 49890613 | T | C | 0.4918 | 0.0168 | 0.0020 | 2.219×10^-17^ | 1.411×10^-4^ | 58.923 |
| rs2535627 | 3 | 52845105 | C | T | 0.4624 | 0.0129 | 0.0020 | 7.423×10^-11^ | 8.279×10^-5^ | 34.575 |
| rs6438900 | 3 | 125148287 | G | C | 0.2580 | 0.0130 | 0.0023 | 1.026×10^-8^ | 6.431×10^-5^ | 26.857 |
| rs6782190 | 3 | 85639672 | A | G | 0.6475 | -0.0189 | 0.0021 | 3.723×10^-20^ | 1.632×10^-4^ | 68.178 |
| rs9861009 | 3 | 141654685 | C | T | 0.7275 | 0.0140 | 0.0022 | 3.227×10^-10^ | 7.746×10^-5^ | 32.347 |
| rs10008500 | 4 | 72059013 | T | A | 0.8769 | -0.0240 | 0.0030 | 1.416×10^-15^ | 1.238×10^-4^ | 51.711 |
| rs11249443 | 4 | 70365831 | A | G | 0.1155 | 0.0173 | 0.0031 | 2.564×10^-8^ | 6.124×10^-5^ | 25.576 |
| rs113256381 | 4 | 73733992 | T | C | 0.0692 | 0.0228 | 0.0039 | 3.892×10^-9^ | 6.702×10^-5^ | 27.987 |
| rs113292111 | 4 | 72795966 | T | C | 0.0122 | 0.0876 | 0.0090 | 2.887×10^-22^ | 1.842×10^-4^ | 76.934 |
| rs114204813 | 4 | 73110572 | G | A | 0.0225 | -0.0815 | 0.0066 | 6.949×10^-35^ | 2.919×10^-4^ | 121.908 |
| rs114687675 | 4 | 72826736 | A | G | 0.0219 | -0.0622 | 0.0067 | 2.323×10^-20^ | 1.652×10^-4^ | 69.013 |
| rs116778432 | 4 | 72360988 | T | C | 0.0171 | 0.0490 | 0.0076 | 1.179×10^-10^ | 8.077×10^-5^ | 33.730 |
| rs11732896 | 4 | 88287993 | A | G | 0.2988 | -0.0150 | 0.0021 | 2.651×10^-12^ | 9.430×10^-5^ | 39.380 |
| rs1229984 | 4 | 100239319 | C | T | 0.9751 | -0.0398 | 0.0063 | 2.357×10^-10^ | 7.701×10^-5^ | 32.159 |
| rs1247583 | 4 | 74489271 | T | C | 0.5413 | -0.0123 | 0.0020 | 5.750×10^-10^ | 7.500×10^-5^ | 31.319 |
| rs12501515 | 4 | 72592838 | A | G | 0.5918 | -0.0713 | 0.0020 | 1.000×10^-200^ | 2.457×10^-3^ | 1028.694 |
| rs12507691 | 4 | 96591676 | A | T | 0.5884 | -0.0113 | 0.0020 | 1.609×10^-8^ | 6.237×10^-5^ | 26.046 |
| rs139959724 | 4 | 72710250 | G | A | 0.0168 | 0.0813 | 0.0077 | 3.913×10^-26^ | 2.181×10^-4^ | 91.093 |
| rs148843488 | 4 | 72474739 | T | G | 0.0252 | 0.0443 | 0.0063 | 2.413×10^-12^ | 9.656×10^-5^ | 40.325 |
| rs150270324 | 4 | 73178175 | C | T | 0.0133 | -0.0672 | 0.0086 | 5.347×10^-15^ | 1.182×10^-4^ | 49.347 |
| rs16846771 | 4 | 72514252 | A | G | 0.1478 | 0.0433 | 0.0028 | 5.338×10^-55^ | 4.722×10^-4^ | 197.276 |
| rs189407772 | 4 | 100146674 | G | A | 0.0227 | 0.0488 | 0.0066 | 1.863×10^-13^ | 1.056×10^-4^ | 44.098 |
| rs2278892 | 4 | 72413746 | G | C | 0.2414 | 0.0208 | 0.0023 | 1.692×10^-19^ | 1.584×10^-4^ | 66.163 |
| rs28855697 | 4 | 100294372 | G | C | 0.1994 | -0.0150 | 0.0025 | 1.413×10^-9^ | 7.191×10^-5^ | 30.028 |
| rs293435 | 4 | 69588052 | T | C | 0.2862 | -0.0175 | 0.0022 | 7.679×10^-16^ | 1.257×10^-4^ | 52.489 |
| rs34760417 | 4 | 72825003 | T | C | 0.0689 | 0.0633 | 0.0039 | 1.160×10^-58^ | 5.151×10^-4^ | 215.191 |
| rs4364259 | 4 | 15892159 | A | G | 0.2022 | 0.0170 | 0.0025 | 5.995×10^-12^ | 9.335×10^-5^ | 38.985 |
| rs4616820 | 4 | 57745481 | T | C | 0.4650 | -0.0125 | 0.0020 | 3.649×10^-10^ | 7.748×10^-5^ | 32.357 |
| rs55814693 | 4 | 87401439 | T | G | 0.2996 | -0.0118 | 0.0022 | 4.318×10^-8^ | 5.824×10^-5^ | 24.320 |
| rs6837680 | 4 | 72735403 | T | A | 0.6570 | 0.0741 | 0.0021 | 1.000×10^-200^ | 2.476×10^-3^ | 1036.550 |
| rs72862854 | 4 | 73533734 | T | C | 0.0638 | -0.0463 | 0.0040 | 1.144×10^-30^ | 2.558×10^-4^ | 106.831 |
| rs7439366 | 4 | 69964338 | C | T | 0.4555 | -0.0294 | 0.0020 | 1.971×10^-50^ | 4.297×10^-4^ | 179.493 |
| rs78649910 | 4 | 3482213 | A | T | 0.1062 | -0.0200 | 0.0032 | 4.744×10^-10^ | 7.579×10^-5^ | 31.651 |
| rs1966478 | 5 | 118627319 | C | T | 0.6930 | -0.0121 | 0.0021 | 1.663×10^-8^ | 6.191×10^-5^ | 25.854 |
| rs9325107 | 5 | 148016857 | T | G | 0.4419 | 0.0112 | 0.0020 | 1.984×10^-8^ | 6.215×10^-5^ | 25.955 |
| rs143069752 | 6 | 40962537 | A | T | 0.0673 | 0.0218 | 0.0039 | 3.079×10^-8^ | 5.968×10^-5^ | 24.922 |
| rs2248551 | 6 | 131924689 | A | G | 0.1652 | -0.0204 | 0.0026 | 1.415×10^-14^ | 1.144×10^-4^ | 47.777 |
| rs35656734 | 6 | 32600153 | T | C | 0.2582 | -0.0134 | 0.0022 | 2.202×10^-9^ | 6.904×10^-5^ | 28.833 |
| rs72834856 | 6 | 22801858 | G | T | 0.0721 | -0.0251 | 0.0038 | 3.705×10^-11^ | 8.450×10^-5^ | 35.290 |
| rs75865451 | 6 | 25588285 | A | G | 0.1041 | -0.0196 | 0.0032 | 9.917×10^-10^ | 7.186×10^-5^ | 30.011 |
| rs9476310 | 6 | 57767576 | T | C | 0.5113 | 0.0109 | 0.0020 | 3.649×10^-8^ | 5.908×10^-5^ | 24.672 |
| rs17144574 | 7 | 21563471 | C | T | 0.2337 | -0.0156 | 0.0023 | 3.172×10^-11^ | 8.666×10^-5^ | 36.190 |
| rs1858889 | 7 | 107117447 | C | A | 0.5015 | 0.0113 | 0.0020 | 8.688×10^-9^ | 6.403×10^-5^ | 26.738 |
| rs2346264 | 7 | 133536351 | C | A | 0.7827 | -0.0143 | 0.0024 | 3.037×10^-9^ | 6.911×10^-5^ | 28.859 |
| rs2528378 | 7 | 43972379 | A | G | 0.3822 | -0.0115 | 0.0020 | 1.605×10^-8^ | 6.250×10^-5^ | 26.100 |
| rs41563 | 7 | 104852654 | A | G | 0.3495 | -0.0118 | 0.0021 | 1.177×10^-8^ | 6.298×10^-5^ | 26.299 |
| rs75419061 | 7 | 64003970 | G | A | 0.0120 | -0.0509 | 0.0091 | 2.163×10^-8^ | 6.122×10^-5^ | 25.567 |
| rs7784802 | 7 | 64015379 | T | A | 0.3610 | 0.0130 | 0.0020 | 2.187×10^-10^ | 7.770×10^-5^ | 32.447 |
| rs12056768 | 8 | 116988527 | G | T | 0.5829 | -0.0216 | 0.0020 | 2.818×10^-27^ | 2.270×10^-4^ | 94.804 |
| rs34726834 | 8 | 25889606 | T | C | 0.2520 | 0.0141 | 0.0023 | 6.231×10^-10^ | 7.476×10^-5^ | 31.221 |
| rs4565433 | 8 | 106497543 | T | G | 0.8462 | 0.0151 | 0.0027 | 3.060×10^-8^ | 5.964×10^-5^ | 24.905 |
| rs57459725 | 8 | 61312205 | G | C | 0.1329 | -0.0160 | 0.0029 | 3.400×10^-8^ | 5.914×10^-5^ | 24.698 |
| rs804281 | 8 | 11611865 | G | A | 0.5836 | 0.0162 | 0.0020 | 4.897×10^-16^ | 1.273×10^-4^ | 53.162 |
| rs13284054 | 9 | 107669073 | C | T | 0.1178 | 0.0178 | 0.0031 | 8.495×10^-9^ | 6.587×10^-5^ | 27.509 |
| rs532436 | 9 | 136149830 | A | G | 0.1838 | -0.0155 | 0.0025 | 8.447×10^-10^ | 7.254×10^-5^ | 30.292 |
| rs9409266 | 9 | 125745042 | A | G | 0.8611 | -0.0176 | 0.0028 | 5.837×10^-10^ | 7.417×10^-5^ | 30.975 |
| rs10887718 | 10 | 82042624 | T | C | 0.5282 | -0.0115 | 0.0020 | 5.853×10^-9^ | 6.565×10^-5^ | 27.417 |
| rs12775091 | 10 | 91524012 | T | C | 0.2144 | 0.0144 | 0.0024 | 1.720×10^-9^ | 6.996×10^-5^ | 29.215 |
| rs4418728 | 10 | 94839724 | T | G | 0.4517 | 0.0112 | 0.0020 | 1.564×10^-8^ | 6.168×10^-5^ | 25.760 |
| rs1011896 | 11 | 15746392 | G | C | 0.5672 | -0.0109 | 0.0020 | 3.773×10^-8^ | 5.831×10^-5^ | 24.351 |
| rs10832164 | 11 | 14048480 | T | C | 0.5172 | 0.0211 | 0.0020 | 1.010×10^-26^ | 2.219×10^-4^ | 92.681 |
| rs10896045 | 11 | 65555524 | G | A | 0.7054 | 0.0123 | 0.0022 | 1.387×10^-8^ | 6.242×10^-5^ | 26.066 |
| rs1149610 | 11 | 76495497 | T | A | 0.1813 | 0.0199 | 0.0026 | 5.968×10^-15^ | 1.181×10^-4^ | 49.335 |
| rs117287238 | 11 | 15090794 | A | G | 0.0261 | 0.0471 | 0.0062 | 3.249×10^-14^ | 1.124×10^-4^ | 46.937 |
| rs117363662 | 11 | 15051809 | A | G | 0.0213 | -0.0915 | 0.0069 | 1.043×10^-40^ | 3.501×10^-4^ | 146.238 |
| rs118055554 | 11 | 14484242 | T | C | 0.0157 | -0.0459 | 0.0079 | 6.279×10^-9^ | 6.504×10^-5^ | 27.162 |
| rs11826004 | 11 | 15132929 | C | T | 0.0618 | 0.0393 | 0.0041 | 6.239×10^-22^ | 1.791×10^-4^ | 74.801 |
| rs142369684 | 11 | 14280765 | A | G | 0.0324 | -0.0604 | 0.0055 | 1.218×10^-27^ | 2.291×10^-4^ | 95.686 |
| rs143488652 | 11 | 13508611 | G | A | 0.0134 | -0.0531 | 0.0086 | 5.634×10^-10^ | 7.457×10^-5^ | 31.143 |
| rs143645388 | 11 | 13350995 | T | C | 0.0165 | -0.0468 | 0.0077 | 1.517×10^-9^ | 7.103×10^-5^ | 29.663 |
| rs1792329 | 11 | 71111182 | T | C | 0.2551 | 0.0258 | 0.0023 | 3.107×10^-29^ | 2.525×10^-4^ | 105.445 |
| rs1792556 | 11 | 15244521 | G | T | 0.3996 | 0.0141 | 0.0020 | 2.319×10^-12^ | 9.603×10^-5^ | 40.104 |
| rs182244780 | 11 | 14385531 | A | G | 0.0130 | -0.3271 | 0.0087 | 1.000×10^-200^ | 2.750×10^-3^ | 1151.663 |
| rs183409297 | 11 | 70547682 | C | T | 0.0256 | 0.0415 | 0.0062 | 2.755×10^-11^ | 8.592×10^-5^ | 35.883 |
| rs1872285 | 11 | 15665052 | A | G | 0.2064 | 0.0164 | 0.0024 | 1.233×10^-11^ | 8.825×10^-5^ | 36.856 |
| rs187706948 | 11 | 15095093 | A | C | 0.0408 | 0.0362 | 0.0050 | 3.455×10^-13^ | 1.027×10^-4^ | 42.878 |
| rs2060793 | 11 | 14915310 | G | A | 0.5955 | -0.0747 | 0.0020 | 1.000×10^-200^ | 2.688×10^-3^ | 1125.665 |
| rs2847500 | 11 | 120114421 | A | G | 0.1235 | -0.0216 | 0.0030 | 5.223×10^-13^ | 1.007×10^-4^ | 42.053 |
| rs55683806 | 11 | 15038424 | A | T | 0.0552 | 0.0247 | 0.0044 | 1.809×10^-8^ | 6.347×10^-5^ | 26.506 |
| rs56019902 | 11 | 14328901 | A | G | 0.0291 | -0.0493 | 0.0058 | 3.231×10^-17^ | 1.373×10^-4^ | 57.327 |
| rs58411334 | 11 | 75467350 | T | G | 0.0931 | 0.0242 | 0.0034 | 8.482×10^-13^ | 9.909×10^-5^ | 41.384 |
| rs61887421 | 11 | 70949673 | C | T | 0.0305 | -0.0321 | 0.0057 | 2.414×10^-8^ | 6.087×10^-5^ | 25.418 |
| rs61891388 | 11 | 66079818 | G | T | 0.4559 | 0.0115 | 0.0020 | 6.300×10^-9^ | 6.571×10^-5^ | 27.440 |
| rs71467497 | 11 | 70471414 | C | T | 0.0286 | -0.0340 | 0.0059 | 9.827×10^-9^ | 6.419×10^-5^ | 26.806 |
| rs72862131 | 11 | 15852030 | G | A | 0.1789 | -0.0171 | 0.0026 | 5.360×10^-11^ | 8.567×10^-5^ | 35.776 |
| rs736894 | 11 | 71152258 | T | C | 0.1825 | -0.0978 | 0.0025 | 1.000×10^-200^ | 2.856×10^-3^ | 1195.857 |
| rs75604577 | 11 | 71033252 | A | C | 0.0205 | -0.0423 | 0.0069 | 1.098×10^-9^ | 7.188×10^-5^ | 30.018 |
| rs77037130 | 11 | 14467795 | A | G | 0.0152 | -0.0609 | 0.0080 | 3.075×10^-14^ | 1.112×10^-4^ | 46.438 |
| rs78168201 | 11 | 70971149 | T | C | 0.0138 | 0.0878 | 0.0085 | 7.954×10^-25^ | 2.093×10^-4^ | 87.436 |
| rs949177 | 11 | 71152461 | G | A | 0.9625 | 0.0963 | 0.0052 | 2.725×10^-77^ | 6.685×10^-4^ | 279.319 |
| rs964184 | 11 | 116648917 | C | G | 0.8684 | 0.0414 | 0.0029 | 4.393×10^-46^ | 3.918×10^-4^ | 163.683 |
| rs9735104 | 11 | 14469365 | T | C | 0.2511 | 0.0548 | 0.0023 | 1.259×10^-127^ | 1.129×10^-3^ | 472.178 |
| rs1038165 | 12 | 68665940 | T | C | 0.5834 | 0.0114 | 0.0020 | 1.040×10^-8^ | 6.316×10^-5^ | 26.376 |
| rs10859995 | 12 | 96375682 | C | T | 0.5826 | -0.0402 | 0.0020 | 1.602×10^-90^ | 7.869×10^-4^ | 328.838 |
| rs10880925 | 12 | 38724296 | C | T | 0.5365 | 0.0123 | 0.0020 | 8.945×10^-10^ | 7.531×10^-5^ | 31.449 |
| rs12307364 | 12 | 96382938 | T | C | 0.2378 | 0.0187 | 0.0023 | 6.705×10^-16^ | 1.269×10^-4^ | 53.009 |
| rs12317268 | 12 | 21352541 | G | A | 0.1510 | -0.0193 | 0.0027 | 2.185×10^-12^ | 9.545×10^-5^ | 39.862 |
| rs73413596 | 12 | 111582630 | C | T | 0.0739 | 0.0220 | 0.0038 | 5.271×10^-9^ | 6.646×10^-5^ | 27.754 |
| rs34284484 | 13 | 55710231 | G | T | 0.2856 | -0.0124 | 0.0022 | 1.201×10^-8^ | 6.263×10^-5^ | 26.155 |
| rs142004400 | 14 | 50829560 | C | A | 0.0345 | -0.0313 | 0.0054 | 6.264×10^-9^ | 6.540×10^-5^ | 27.311 |
| rs2756119 | 14 | 104001517 | A | G | 0.3833 | 0.0119 | 0.0020 | 5.246×10^-9^ | 6.744×10^-5^ | 28.164 |
| rs8018720 | 14 | 39556185 | C | G | 0.8233 | -0.0300 | 0.0026 | 2.633×10^-31^ | 2.611×10^-4^ | 109.074 |
| rs1800588 | 15 | 58723675 | T | C | 0.2152 | -0.0308 | 0.0024 | 4.273×10^-38^ | 3.212×10^-4^ | 134.152 |
| rs2123930 | 15 | 100231033 | A | G | 0.2794 | -0.0128 | 0.0022 | 5.666×10^-9^ | 6.620×10^-5^ | 27.646 |
| rs261291 | 15 | 58680178 | C | T | 0.3552 | -0.0256 | 0.0021 | 1.233×10^-35^ | 3.012×10^-4^ | 125.799 |
| rs55829990 | 15 | 63790642 | C | T | 0.3440 | -0.0192 | 0.0021 | 2.260×10^-20^ | 1.662×10^-4^ | 69.428 |
| rs58038553 | 15 | 58574324 | G | A | 0.1285 | 0.0192 | 0.0030 | 7.234×10^-11^ | 8.285×10^-5^ | 34.601 |
| rs10083762 | 16 | 11907210 | G | C | 0.2727 | 0.0121 | 0.0022 | 4.032×10^-8^ | 5.833×10^-5^ | 24.359 |
| rs11076175 | 16 | 57006378 | G | A | 0.1784 | 0.0227 | 0.0026 | 1.268×10^-18^ | 1.505×10^-4^ | 62.844 |
| rs11542462 | 16 | 82033810 | A | G | 0.1343 | -0.0237 | 0.0029 | 1.918×10^-16^ | 1.305×10^-4^ | 54.498 |
| rs4575545 | 16 | 79755446 | A | G | 0.3048 | -0.0146 | 0.0021 | 1.009×10^-11^ | 9.024×10^-5^ | 37.684 |
| rs55872725 | 16 | 53809123 | T | C | 0.4033 | 0.0112 | 0.0020 | 2.496×10^-8^ | 5.998×10^-5^ | 25.046 |
| rs12949853 | 17 | 7570878 | A | G | 0.8067 | 0.0140 | 0.0025 | 3.442×10^-8^ | 6.123×10^-5^ | 25.569 |
| rs2659007 | 17 | 79217478 | A | G | 0.4510 | 0.0109 | 0.0020 | 4.943×10^-8^ | 5.854×10^-5^ | 24.447 |
| rs2952289 | 17 | 66464414 | T | C | 0.7980 | 0.0159 | 0.0025 | 9.378×10^-11^ | 8.177×10^-5^ | 34.148 |
| rs2037511 | 18 | 61366207 | A | G | 0.1660 | 0.0169 | 0.0026 | 1.824×10^-10^ | 7.868×10^-5^ | 32.859 |
| rs7244811 | 18 | 47156730 | A | G | 0.7726 | -0.0148 | 0.0024 | 4.491×10^-10^ | 7.709×10^-5^ | 32.193 |
| rs77960347 | 18 | 47109955 | G | A | 0.0131 | -0.0504 | 0.0086 | 5.084×10^-9^ | 6.582×10^-5^ | 27.488 |
| rs8091117 | 18 | 28919794 | A | C | 0.0653 | -0.0242 | 0.0040 | 1.162×10^-9^ | 7.142×10^-5^ | 29.824 |
| rs12462826 | 19 | 11955767 | A | G | 0.3687 | -0.0127 | 0.0021 | 6.483×10^-10^ | 7.487×10^-5^ | 31.268 |
| rs142158911 | 19 | 11190534 | A | G | 0.1146 | 0.0272 | 0.0031 | 2.036×10^-18^ | 1.499×10^-4^ | 62.590 |
| rs188247550 | 19 | 19396616 | T | C | 0.0112 | 0.0587 | 0.0094 | 4.009×10^-10^ | 7.621×10^-5^ | 31.826 |
| rs212100 | 19 | 48376995 | C | T | 0.8360 | -0.0603 | 0.0027 | 4.436×10^-114^ | 9.956×10^-4^ | 416.135 |
| rs3814995 | 19 | 36342212 | T | C | 0.3116 | -0.0125 | 0.0021 | 3.605×10^-9^ | 6.723×10^-5^ | 28.075 |
| rs6857 | 19 | 45392254 | T | C | 0.1686 | -0.0212 | 0.0026 | 6.568×10^-16^ | 1.261×10^-4^ | 52.676 |
| rs7248342 | 19 | 51515549 | G | A | 0.2358 | 0.0232 | 0.0023 | 2.328×10^-23^ | 1.948×10^-4^ | 81.357 |
| rs7412 | 19 | 45412079 | T | C | 0.0821 | 0.0326 | 0.0036 | 9.972×10^-20^ | 1.601×10^-4^ | 66.854 |
| rs8107974 | 19 | 19388500 | T | A | 0.0762 | 0.0358 | 0.0037 | 3.693×10^-22^ | 1.810×10^-4^ | 75.614 |
| rs139415780 | 20 | 52747748 | A | G | 0.1466 | -0.0224 | 0.0028 | 6.945×10^-16^ | 1.261×10^-4^ | 52.650 |
| rs140433285 | 20 | 52776021 | T | C | 0.0324 | -0.0345 | 0.0056 | 8.339×10^-10^ | 7.458×10^-5^ | 31.146 |
| rs2207132 | 20 | 39142516 | A | G | 0.0329 | -0.0337 | 0.0055 | 8.863×10^-10^ | 7.237×10^-5^ | 30.223 |
| rs2585442 | 20 | 52737123 | G | C | 0.2407 | 0.0342 | 0.0023 | 3.142×10^-48^ | 4.280×10^-4^ | 178.819 |
| rs2762943 | 20 | 52790786 | G | T | 0.9231 | 0.0453 | 0.0037 | 1.144×10^-34^ | 2.914×10^-4^ | 121.708 |
| rs3787557 | 20 | 52783135 | C | T | 0.1289 | 0.0193 | 0.0030 | 8.000×10^-11^ | 8.337×10^-5^ | 34.818 |
| rs6011153 | 20 | 62450502 | C | G | 0.0627 | 0.0230 | 0.0040 | 1.400×10^-8^ | 6.204×10^-5^ | 25.910 |
| rs6123359 | 20 | 52714706 | G | A | 0.1022 | 0.0318 | 0.0033 | 2.044×10^-22^ | 1.862×10^-4^ | 77.765 |
| rs8114057 | 20 | 43036452 | A | G | 0.4636 | 0.0111 | 0.0020 | 1.846×10^-8^ | 6.166×10^-5^ | 25.749 |
| rs2229742 | 21 | 16339172 | C | G | 0.1035 | -0.0240 | 0.0032 | 9.238×10^-14^ | 1.073×10^-4^ | 44.798 |
| rs2074735 | 22 | 31535872 | C | G | 0.0641 | 0.0275 | 0.0040 | 7.607×10^-12^ | 9.068×10^-5^ | 37.871 |
| rs5770982 | 22 | 50865434 | G | A | 0.3219 | -0.0115 | 0.0021 | 4.013×10^-8^ | 5.814×10^-5^ | 24.280 |

**Abbreviations:** EAF, effect allele frequency; SE, standard error; SNP, single nucleotide polymorphism.
