# Supplementary material for: A Causal Effect of Serum 25(OH)D Level on Appendicular Muscle Mass: Evidence From NHANES Data and Mendelian Randomization Analyses
Source: J Cachexia Sarcopenia Muscle. 2025 Mar 31;16(2):e13778. doi: 10.1002/jcsm.13778 (PMC11955837; doi:10.1002/jcsm.13778)
Supplement: Supplementary file 3 — Table S3. Multivariate linear regression analysis of the associations between vitamin D status and appendicular lean mass index in male (n = 5588) and female (n = 5654) participants from NHANES 2011–2018. [file JCSM-16-e13778-s006.docx]

**Supplementary Table 3.** Multivariate linear regression analysis of the associations between vitamin D status and appendicular lean mass index in male (n=5,588) and female (n=5,654) participants from NHANES 2011–2018.

| Appendicular lean mass index | Total (n = 11,242) | | Male (n = 5,588) | | Female (n = 5,654) | |
| --- | --- | --- | --- | --- | --- | --- |
|  | *β (SE)* | *P* value | *β (SE)* | *P* value | *β (SE)* | *P* value |
| 25-hydroxyvitamin D, nM | 0.003 (3.686×10^-4^) | < 0.001 | 0.006 (0.001) | < 0.001 | 0.001 (4.409×10^-4^) | 0.017 |
| Age, years | -0.017 (0.001) | < 0.001 | -0.017 (0.001) | < 0.001 | -0.017 (0.001) | < 0.001 |
| Sex (female *vs.* male) | -1.971 (0.016) | < 0.001 | — | | — | |
| Race |  |  |  |  |  |  |
| Mexican American | Reference |  | Reference |  | Reference |  |
| Other Hispanic | 0.163 (0.033) | < 0.001 | 0.159 (0.051) | 0.002 | 0.167 (0.042) | < 0.001 |
| Non-Hispanic White | 0.001 (0.027) | 0.969 | -0.094 (0.040) | 0.019 | 0.112 (0.035) | 0.001 |
| Non-Hispanic Black | 0.808 (0.029) | < 0.001 | 0.891 (0.043) | < 0.001 | 0.766 (0.037) | < 0.001 |
| Other Races | 0.063 (0.030) | 0.033 | 0.020 (0.044) | 0.655 | 0.127 (0.039) | 0.001 |
| Season (Spring & Summer *vs.* Autumn & Winter) | 0.009 (0.017) | 0.601 | 0.007 (0.025) | 0.769 | 0.008 (0.021) | 0.701 |
| Education |  |  |  |  |  |  |
| Less than high school | Reference |  | Reference |  | Reference |  |
| High school or equivalent | -0.031 (0.026) | 0.232 | -0.060 (0.037) | 0.109 | -0.002 (0.035) | 0.957 |
| College or above | 0.097 (0.024) | < 0.001 | 0.075 (0.035) | 0.035 | 0.121 (0.032) | < 0.001 |
| Poverty index ratios |  |  |  |  |  |  |
| ≤1.0 | Reference |  | Reference |  | Reference |  |
| 1.0 to 3.0 | 0.068 (0.021) | 0.001 | 0.071 (0.032) | 0.029 | 0.052 (0.027) | 0.056 |
| >3.0 | 0.117 (0.024) | < 0.001 | 0.130 (0.036) | < 0.001 | 0.080 (0.030) | 0.008 |
| Body mass index, kg/m^2^ | 0.181 (0.001) | < 0.001 | 0.199 (0.002) | < 0.001 | 0.169 (0.001) | < 0.001 |
| Sedentary time, h/d | -0.016 (0.002) | < 0.001 | -0.019 (0.004) | < 0.001 | -0.012 (0.003) | < 0.001 |

SE, standard error. The multivariate linear regression model was adjusted for age, race, the month of blood collection, education, poverty index ratio, body mass index and sedentary time.
