# Supplementary material for: A Causal Effect of Serum 25(OH)D Level on Appendicular Muscle Mass: Evidence From NHANES Data and Mendelian Randomization Analyses
Source: J Cachexia Sarcopenia Muscle. 2025 Mar 31;16(2):e13778. doi: 10.1002/jcsm.13778 (PMC11955837; doi:10.1002/jcsm.13778)
Supplement: Supplementary file 4 — Table S4. The pleiotropy effects for the genetic instrumental variables in the two‐sample Mendelian randomization studies to evaluate whether the causality estimate in the total population was affected by a single SNP using leave‐one‐out methods based on conventional inverse variance weighted model. [file JCSM-16-e13778-s002.docx]

**Supplementary Table 4**. The pleiotropy effects for the genetic instrumental variables in the two-sample Mendelian randomization studies to evaluate whether the causality estimate in the total population was affected by a single SNP using leave-one-out methods based on conventional inverse variance weighted model.

| All | Sample size | SNP | *β* | *SE* | *P* |
| --- | --- | --- | --- | --- | --- |
| 1 | 450243 | rs10008500 | 0.048527624 | 0.023664677 | 0.040302468 |
| 2 | 450243 | rs10083762 | 0.048494148 | 0.023643305 | 0.040259829 |
| 3 | 450243 | rs1038165 | 0.048079968 | 0.023624049 | 0.041829459 |
| 4 | 450243 | rs1047891 | 0.052190789 | 0.022716138 | 0.021589104 |
| 5 | 450243 | rs10832164 | 0.049330516 | 0.023686665 | 0.037285125 |
| 6 | 450243 | rs10859995 | 0.050396838 | 0.02387161 | 0.034758382 |
| 7 | 450243 | rs10880925 | 0.048311799 | 0.023643381 | 0.041017404 |
| 8 | 450243 | rs10887718 | 0.048999691 | 0.023636763 | 0.038169595 |
| 9 | 450243 | rs10896045 | 0.049641881 | 0.023565767 | 0.035158766 |
| 10 | 450243 | rs10908465 | 0.049291418 | 0.023637388 | 0.037040651 |
| 11 | 450243 | rs11076175 | 0.046021081 | 0.023453438 | 0.049735508 |
| 12 | 450243 | rs11127186 | 0.048606566 | 0.023645188 | 0.039814898 |
| 13 | 450243 | rs11204743 | 0.050714459 | 0.023443629 | 0.03052186 |
| 14 | 450243 | rs11249443 | 0.048837667 | 0.023642956 | 0.038863157 |
| 15 | 450243 | rs11264223 | 0.047038692 | 0.023459679 | 0.044953774 |
| 16 | 450243 | rs113256381 | 0.049074228 | 0.02363382 | 0.037853201 |
| 17 | 450243 | rs113292111 | 0.050030001 | 0.023660964 | 0.034476576 |
| 18 | 450243 | rs114204813 | 0.048193782 | 0.023716895 | 0.042149165 |
| 19 | 450243 | rs114687675 | 0.048153275 | 0.023673963 | 0.041949741 |
| 20 | 450243 | rs1149610 | 0.04989981 | 0.023595584 | 0.034447471 |
| 21 | 450243 | rs11542462 | 0.048891678 | 0.023664533 | 0.03882548 |
| 22 | 450243 | rs11591147 | 0.049420914 | 0.023607023 | 0.036306016 |
| 23 | 450243 | rs116778432 | 0.048197498 | 0.023641578 | 0.041482455 |
| 24 | 450243 | rs117287238 | 0.049262668 | 0.023645825 | 0.037218801 |
| 25 | 450243 | rs11732896 | 0.0473796 | 0.023573933 | 0.044449177 |
| 26 | 450243 | rs117363662 | 0.049300551 | 0.023743265 | 0.037856837 |
| 27 | 450243 | rs118055554 | 0.048608544 | 0.023646528 | 0.039818065 |
| 28 | 450243 | rs11826004 | 0.047280687 | 0.023637097 | 0.045470607 |
| 29 | 450243 | rs12056768 | 0.047403349 | 0.023670071 | 0.045212683 |
| 30 | 450243 | rs12123821 | 0.052892767 | 0.023650187 | 0.025321459 |
| 31 | 450243 | rs1229984 | 0.048289031 | 0.023646295 | 0.041137706 |
| 32 | 450243 | rs12307364 | 0.049013839 | 0.023662149 | 0.038321176 |
| 33 | 450243 | rs12317268 | 0.047849036 | 0.023628116 | 0.042858231 |
| 34 | 450243 | rs12462826 | 0.048883068 | 0.023644599 | 0.038695589 |
| 35 | 450243 | rs1247583 | 0.048965497 | 0.023642653 | 0.038352622 |
| 36 | 450243 | rs12501515 | 0.048910484 | 0.024487752 | 0.04578774 |
| 37 | 450243 | rs12507691 | 0.048035673 | 0.023619896 | 0.041982317 |
| 38 | 450243 | rs1260326 | 0.039808721 | 0.02187889 | 0.068834626 |
| 39 | 450243 | rs12775091 | 0.048148344 | 0.023632556 | 0.041612629 |
| 40 | 450243 | rs12949853 | 0.046223002 | 0.023191629 | 0.046251602 |
| 41 | 450243 | rs13284054 | 0.048564753 | 0.023647012 | 0.040001117 |
| 42 | 450243 | rs139415780 | 0.048174297 | 0.023657661 | 0.041719387 |
| 43 | 450243 | rs139959724 | 0.047231437 | 0.023659351 | 0.045900018 |
| 44 | 450243 | rs140371183 | 0.051105259 | 0.023703486 | 0.031081879 |
| 45 | 450243 | rs140433285 | 0.048374719 | 0.023648225 | 0.040796123 |
| 46 | 450243 | rs142004400 | 0.049788767 | 0.023548616 | 0.034489913 |
| 47 | 450243 | rs142158911 | 0.047517371 | 0.023633253 | 0.044366153 |
| 48 | 450243 | rs142369684 | 0.050187382 | 0.023652028 | 0.033845621 |
| 49 | 450243 | rs143069752 | 0.04811071 | 0.023624975 | 0.041706761 |
| 50 | 450243 | rs143488652 | 0.049355537 | 0.023618155 | 0.036642174 |
| 51 | 450243 | rs143645388 | 0.048526048 | 0.023648553 | 0.040172878 |
| 52 | 450243 | rs148843488 | 0.049530008 | 0.023622794 | 0.036019781 |
| 53 | 450243 | rs150270324 | 0.050135575 | 0.023575229 | 0.033451593 |
| 54 | 450243 | rs16846771 | 0.047982503 | 0.023771735 | 0.043542475 |
| 55 | 450243 | rs17144574 | 0.0481334 | 0.023641401 | 0.041752444 |
| 56 | 450243 | rs1792329 | 0.049461197 | 0.023692216 | 0.036828875 |
| 57 | 450243 | rs1792556 | 0.049284673 | 0.023637278 | 0.037065659 |
| 58 | 450243 | rs1800588 | 0.048515139 | 0.023730191 | 0.04090932 |
| 59 | 450243 | rs182244780 | 0.055475893 | 0.02450508 | 0.023583174 |
| 60 | 450243 | rs183409297 | 0.048508616 | 0.023653382 | 0.040285351 |
| 61 | 450243 | rs1858889 | 0.049224636 | 0.023619753 | 0.03715604 |
| 62 | 450243 | rs1872285 | 0.049684664 | 0.023597369 | 0.035246323 |
| 63 | 450243 | rs187706948 | 0.049217281 | 0.023644653 | 0.037384546 |
| 64 | 450243 | rs188247550 | 0.048346638 | 0.023647168 | 0.040904164 |
| 65 | 450243 | rs189407772 | 0.04870415 | 0.023661201 | 0.039552019 |
| 66 | 450243 | rs1966478 | 0.047842472 | 0.023601373 | 0.042651631 |
| 67 | 450243 | rs2012736 | 0.048223729 | 0.023727605 | 0.042114262 |
| 68 | 450243 | rs2037511 | 0.049210106 | 0.023628756 | 0.037284386 |
| 69 | 450243 | rs2060793 | 0.045917317 | 0.024564666 | 0.061589085 |
| 70 | 450243 | rs2074735 | 0.046844841 | 0.02348433 | 0.046072603 |
| 71 | 450243 | rs212100 | 0.048914183 | 0.023942925 | 0.04105745 |
| 72 | 450243 | rs2123930 | 0.047174876 | 0.023496436 | 0.044670286 |
| 73 | 450243 | rs2131925 | 0.049811685 | 0.023657448 | 0.035244391 |
| 74 | 450243 | rs2207132 | 0.048184097 | 0.023635932 | 0.041490441 |
| 75 | 450243 | rs2229742 | 0.048343546 | 0.02365678 | 0.040999137 |
| 76 | 450243 | rs2248551 | 0.049432147 | 0.023638468 | 0.036512477 |
| 77 | 450243 | rs2278892 | 0.047446201 | 0.023635972 | 0.044710016 |
| 78 | 450243 | rs2346264 | 0.048855296 | 0.023644263 | 0.038803531 |
| 79 | 450243 | rs2352974 | 0.048198757 | 0.023664608 | 0.041675667 |
| 80 | 450243 | rs2528378 | 0.048922373 | 0.02364059 | 0.03850644 |
| 81 | 450243 | rs2535627 | 0.047280711 | 0.023547654 | 0.044656734 |
| 82 | 450243 | rs2585442 | 0.049075145 | 0.023764927 | 0.038920724 |
| 83 | 450243 | rs261291 | 0.048709197 | 0.023721262 | 0.040033973 |
| 84 | 450243 | rs2659007 | 0.048723973 | 0.023643739 | 0.039326339 |
| 85 | 450243 | rs2710647 | 0.049340239 | 0.023609637 | 0.036632651 |
| 86 | 450243 | rs2756119 | 0.047467391 | 0.023545679 | 0.043803073 |
| 87 | 450243 | rs2762943 | 0.048198788 | 0.023714687 | 0.042108664 |
| 88 | 450243 | rs2847500 | 0.048523312 | 0.023657016 | 0.040255539 |
| 89 | 450243 | rs28855697 | 0.049185982 | 0.023627312 | 0.037365988 |
| 90 | 450243 | rs293435 | 0.047132018 | 0.023580836 | 0.045636223 |
| 91 | 450243 | rs2952289 | 0.047062191 | 0.023503002 | 0.045242736 |
| 92 | 450243 | rs34284484 | 0.048893759 | 0.023640387 | 0.038618391 |
| 93 | 450243 | rs34726834 | 0.04868591 | 0.023649026 | 0.039524365 |
| 94 | 450243 | rs34760417 | 0.050378843 | 0.023769972 | 0.034053959 |
| 95 | 450243 | rs35285316 | 0.048630135 | 0.023644342 | 0.039711769 |
| 96 | 450243 | rs35408430 | 0.050274065 | 0.023622105 | 0.033315234 |
| 97 | 450243 | rs35656734 | 0.047485077 | 0.023564198 | 0.043890339 |
| 98 | 450243 | rs3787557 | 0.048405426 | 0.023650099 | 0.040684353 |
| 99 | 450243 | rs3814995 | 0.048277948 | 0.023639785 | 0.041128427 |
| 100 | 450243 | rs41563 | 0.048207046 | 0.023631825 | 0.041358241 |
| 101 | 450243 | rs4364259 | 0.04841666 | 0.023652209 | 0.040655643 |
| 102 | 450243 | rs4418728 | 0.04903272 | 0.023633084 | 0.038009794 |
| 103 | 450243 | rs4565433 | 0.048579713 | 0.023644904 | 0.039922184 |
| 104 | 450243 | rs4575545 | 0.047394364 | 0.023569721 | 0.044344933 |
| 105 | 450243 | rs4616820 | 0.046624338 | 0.023402725 | 0.046342423 |
| 106 | 450243 | rs512083 | 0.049979364 | 0.023529841 | 0.033663103 |
| 107 | 450243 | rs532436 | 0.048531987 | 0.023649191 | 0.040153853 |
| 108 | 450243 | rs541041 | 0.048935224 | 0.023642713 | 0.038472917 |
| 109 | 450243 | rs55683806 | 0.048774985 | 0.023646081 | 0.039140279 |
| 110 | 450243 | rs55707527 | 0.045515271 | 0.023054584 | 0.048354515 |
| 111 | 450243 | rs55814693 | 0.047418405 | 0.023519969 | 0.043790429 |
| 112 | 450243 | rs55829990 | 0.049538216 | 0.023655422 | 0.036245816 |
| 113 | 450243 | rs55872725 | 0.045336036 | 0.022799702 | 0.046762096 |
| 114 | 450243 | rs56019902 | 0.048467856 | 0.023669609 | 0.040591076 |
| 115 | 450243 | rs57459725 | 0.048678956 | 0.02364424 | 0.039512616 |
| 116 | 450243 | rs5770982 | 0.049122077 | 0.023625604 | 0.037600052 |
| 117 | 450243 | rs58038553 | 0.049504524 | 0.023610067 | 0.036015274 |
| 118 | 450243 | rs58387006 | 0.049962609 | 0.023507689 | 0.033555501 |
| 119 | 450243 | rs58411334 | 0.048913153 | 0.023652824 | 0.038643553 |
| 120 | 450243 | rs6011153 | 0.047621536 | 0.023567894 | 0.043320095 |
| 121 | 450243 | rs6123359 | 0.049113301 | 0.023681077 | 0.038084226 |
| 122 | 450243 | rs61887421 | 0.049088734 | 0.023630221 | 0.037767337 |
| 123 | 450243 | rs61891388 | 0.048169602 | 0.023631412 | 0.04151277 |
| 124 | 450243 | rs6438900 | 0.049095906 | 0.023629109 | 0.037730345 |
| 125 | 450243 | rs6671730 | 0.048702606 | 0.023650923 | 0.03947255 |
| 126 | 450243 | rs6672758 | 0.04725773 | 0.023525547 | 0.044560099 |
| 127 | 450243 | rs6724965 | 0.048902435 | 0.023646009 | 0.038630133 |
| 128 | 450243 | rs6782190 | 0.045953355 | 0.023464993 | 0.050185536 |
| 129 | 450243 | rs6837680 | 0.054197847 | 0.024407276 | 0.026380706 |
| 130 | 450243 | rs6857 | 0.049472576 | 0.023641318 | 0.036382027 |
| 131 | 450243 | rs71467497 | 0.049125633 | 0.023630642 | 0.037626964 |
| 132 | 450243 | rs7244811 | 0.049198558 | 0.023628976 | 0.037330755 |
| 133 | 450243 | rs7248342 | 0.049444645 | 0.023674837 | 0.036753715 |
| 134 | 450243 | rs72834856 | 0.050170391 | 0.023525044 | 0.032954474 |
| 135 | 450243 | rs72862131 | 0.049226649 | 0.023632423 | 0.037250019 |
| 136 | 450243 | rs72862854 | 0.044019893 | 0.023306111 | 0.058922599 |
| 137 | 450243 | rs73413596 | 0.047189247 | 0.023500283 | 0.044640226 |
| 138 | 450243 | rs7367758 | 0.046392084 | 0.023346954 | 0.046914393 |
| 139 | 450243 | rs736894 | 0.055948221 | 0.024570737 | 0.02278464 |
| 140 | 450243 | rs7412 | 0.048891253 | 0.023673812 | 0.03890369 |
| 141 | 450243 | rs7439366 | 0.048643472 | 0.023765863 | 0.040679776 |
| 142 | 450243 | rs7528419 | 0.045403403 | 0.023297491 | 0.051312889 |
| 143 | 450243 | rs75419061 | 0.048114696 | 0.023630496 | 0.041737579 |
| 144 | 450243 | rs75604577 | 0.049259007 | 0.02362235 | 0.037044688 |
| 145 | 450243 | rs7569755 | 0.04935325 | 0.023616869 | 0.036640653 |
| 146 | 450243 | rs75865451 | 0.049812371 | 0.023556575 | 0.034465367 |
| 147 | 450243 | rs7604788 | 0.049259965 | 0.023629279 | 0.037096516 |
| 148 | 450243 | rs7784802 | 0.048029013 | 0.023627155 | 0.042073875 |
| 149 | 450243 | rs77960347 | 0.048215143 | 0.023634445 | 0.041346636 |
| 150 | 450243 | rs78168201 | 0.048739523 | 0.023707329 | 0.03979368 |
| 151 | 450243 | rs78649910 | 0.046737031 | 0.023436776 | 0.046132946 |
| 152 | 450243 | rs78886843 | 0.049043585 | 0.023639401 | 0.038018582 |
| 153 | 450243 | rs8018720 | 0.048098978 | 0.02370399 | 0.042442895 |
| 154 | 450243 | rs804281 | 0.047412622 | 0.023613583 | 0.044658832 |
| 155 | 450243 | rs8091117 | 0.048818524 | 0.023646674 | 0.038970467 |
| 156 | 450243 | rs8107974 | 0.052322906 | 0.023317841 | 0.024838828 |
| 157 | 450243 | rs8114057 | 0.048834369 | 0.023642179 | 0.038869923 |
| 158 | 450243 | rs867772 | 0.047167103 | 0.023551988 | 0.045211612 |
| 159 | 450243 | rs9325107 | 0.048362462 | 0.023640243 | 0.040779167 |
| 160 | 450243 | rs9409266 | 0.047341606 | 0.023546729 | 0.044374218 |
| 161 | 450243 | rs9476310 | 0.048116493 | 0.023623092 | 0.041665937 |
| 162 | 450243 | rs949177 | 0.049369737 | 0.023848865 | 0.038442189 |
| 163 | 450243 | rs964184 | 0.049080014 | 0.023750862 | 0.038785831 |
| 164 | 450243 | rs9735104 | 0.050274328 | 0.023982562 | 0.036056761 |
| 165 | 450243 | rs9861009 | 0.048799266 | 0.023649471 | 0.039070834 |
| 166 | 450243 | All | 0.048630812 | 0.023553277 | 0.038949946 |

**Abbreviations:** SE, standard error; SNP, single nucleotide polymorphism.
