# Supplementary material for: A Causal Effect of Serum 25(OH)D Level on Appendicular Muscle Mass: Evidence From NHANES Data and Mendelian Randomization Analyses
Source: J Cachexia Sarcopenia Muscle. 2025 Mar 31;16(2):e13778. doi: 10.1002/jcsm.13778 (PMC11955837; doi:10.1002/jcsm.13778)
Supplement: Supplementary file 5 — Table S5. The pleiotropy effects for the genetic instrumental variables in the two‐sample Mendelian randomization studies to evaluate whether the causality estimate in males was affected by a single SNP using leave‐one‐out methods based on conventional inverse variance weighted model. [file JCSM-16-e13778-s001.docx]

**Supplementary Table 5**. The pleiotropy effects for the genetic instrumental variables in the two-sample Mendelian randomization studies to evaluate whether the causality estimate in males was affected by a single SNP using leave-one-out methods based on conventional inverse variance weighted model.

| Males | Sample size | SNP | *β* | *SE* | *P* |
| --- | --- | --- | --- | --- | --- |
| 1 | 205513 | rs10008500 | 0.057240033 | 0.024631435 | 0.020132943 |
| 2 | 205513 | rs10083762 | 0.056711906 | 0.024619764 | 0.021250089 |
| 3 | 205513 | rs1038165 | 0.056568381 | 0.02462072 | 0.021584996 |
| 4 | 205513 | rs1047891 | 0.059347433 | 0.024122588 | 0.013884289 |
| 5 | 205513 | rs10832164 | 0.05789395 | 0.024645369 | 0.018819849 |
| 6 | 205513 | rs10859995 | 0.059825925 | 0.024818419 | 0.015928691 |
| 7 | 205513 | rs10880925 | 0.056514066 | 0.024623587 | 0.021726299 |
| 8 | 205513 | rs10887718 | 0.057213836 | 0.024602987 | 0.02004613 |
| 9 | 205513 | rs10896045 | 0.057473322 | 0.02457593 | 0.019356033 |
| 10 | 205513 | rs10908465 | 0.056627163 | 0.024633291 | 0.021515946 |
| 11 | 205513 | rs11076175 | 0.053342361 | 0.024312052 | 0.028230328 |
| 12 | 205513 | rs11127186 | 0.056210811 | 0.024604597 | 0.022338326 |
| 13 | 205513 | rs11204743 | 0.058980287 | 0.024389299 | 0.015593837 |
| 14 | 205513 | rs11249443 | 0.0568853 | 0.024618053 | 0.020848614 |
| 15 | 205513 | rs11264223 | 0.0553559 | 0.024502843 | 0.023873288 |
| 16 | 205513 | rs113256381 | 0.056771823 | 0.024622549 | 0.021128282 |
| 17 | 205513 | rs113292111 | 0.057567139 | 0.024663032 | 0.019588034 |
| 18 | 205513 | rs114204813 | 0.056402994 | 0.024697113 | 0.022383935 |
| 19 | 205513 | rs114687675 | 0.05528532 | 0.024605536 | 0.024648648 |
| 20 | 205513 | rs1149610 | 0.058104569 | 0.024563161 | 0.018004928 |
| 21 | 205513 | rs11542462 | 0.056309152 | 0.02463963 | 0.022294755 |
| 22 | 205513 | rs11591147 | 0.0575776 | 0.024572443 | 0.019120278 |
| 23 | 205513 | rs116778432 | 0.0556264 | 0.024568339 | 0.023564906 |
| 24 | 205513 | rs117287238 | 0.057244128 | 0.024626923 | 0.02010122 |
| 25 | 205513 | rs11732896 | 0.055389298 | 0.024553027 | 0.024076579 |
| 26 | 205513 | rs117363662 | 0.057257787 | 0.024725655 | 0.020573358 |
| 27 | 205513 | rs118055554 | 0.056879247 | 0.024619089 | 0.020867582 |
| 28 | 205513 | rs11826004 | 0.055121035 | 0.024600096 | 0.025046565 |
| 29 | 205513 | rs12056768 | 0.055590345 | 0.024653604 | 0.024142322 |
| 30 | 205513 | rs12123821 | 0.061023055 | 0.024632417 | 0.0132363 |
| 31 | 205513 | rs1229984 | 0.056451655 | 0.024626279 | 0.021886382 |
| 32 | 205513 | rs12307364 | 0.057138687 | 0.024636078 | 0.020378264 |
| 33 | 205513 | rs12317268 | 0.056000287 | 0.024611298 | 0.022882581 |
| 34 | 205513 | rs12462826 | 0.056624824 | 0.024624859 | 0.021476653 |
| 35 | 205513 | rs1247583 | 0.056836117 | 0.024624066 | 0.020990616 |
| 36 | 205513 | rs12501515 | 0.056509559 | 0.025479844 | 0.026567503 |
| 37 | 205513 | rs12507691 | 0.056313259 | 0.024611354 | 0.022131652 |
| 38 | 205513 | rs1260326 | 0.048305736 | 0.023101336 | 0.036524792 |
| 39 | 205513 | rs12775091 | 0.056067692 | 0.024599402 | 0.022653401 |
| 40 | 205513 | rs12949853 | 0.054152103 | 0.024145464 | 0.024913305 |
| 41 | 205513 | rs13284054 | 0.056937595 | 0.024618159 | 0.020732037 |
| 42 | 205513 | rs139415780 | 0.055865323 | 0.024619265 | 0.023257929 |
| 43 | 205513 | rs139959724 | 0.055711373 | 0.024659156 | 0.023867398 |
| 44 | 205513 | rs140371183 | 0.060372098 | 0.024615798 | 0.01418377 |
| 45 | 205513 | rs140433285 | 0.056850323 | 0.02462601 | 0.020968685 |
| 46 | 205513 | rs142004400 | 0.057791648 | 0.024537289 | 0.018509845 |
| 47 | 205513 | rs142158911 | 0.055212758 | 0.024585006 | 0.024717469 |
| 48 | 205513 | rs142369684 | 0.057800606 | 0.024654314 | 0.019055581 |
| 49 | 205513 | rs143069752 | 0.056648582 | 0.024620541 | 0.021399166 |
| 50 | 205513 | rs143488652 | 0.057434995 | 0.024593499 | 0.01952394 |
| 51 | 205513 | rs143645388 | 0.056660992 | 0.024625323 | 0.021395938 |
| 52 | 205513 | rs148843488 | 0.057523234 | 0.024605672 | 0.019397428 |
| 53 | 205513 | rs150270324 | 0.058194013 | 0.024555276 | 0.017791989 |
| 54 | 205513 | rs16846771 | 0.056442332 | 0.024762184 | 0.022644713 |
| 55 | 205513 | rs17144574 | 0.056763223 | 0.024629034 | 0.021181813 |
| 56 | 205513 | rs1792329 | 0.056726542 | 0.02468491 | 0.021560364 |
| 57 | 205513 | rs1792556 | 0.057332455 | 0.024612493 | 0.019837642 |
| 58 | 205513 | rs1800588 | 0.055300429 | 0.024683211 | 0.025064536 |
| 59 | 205513 | rs182244780 | 0.062791339 | 0.025507239 | 0.013827775 |
| 60 | 205513 | rs183409297 | 0.056261525 | 0.024620871 | 0.022306077 |
| 61 | 205513 | rs1858889 | 0.057402452 | 0.024585341 | 0.019552598 |
| 62 | 205513 | rs1872285 | 0.057004379 | 0.024624743 | 0.020617158 |
| 63 | 205513 | rs187706948 | 0.057023891 | 0.024630861 | 0.020605276 |
| 64 | 205513 | rs188247550 | 0.056329244 | 0.024620886 | 0.022145429 |
| 65 | 205513 | rs189407772 | 0.056439113 | 0.024634448 | 0.021959675 |
| 66 | 205513 | rs1966478 | 0.055885151 | 0.024575248 | 0.02296345 |
| 67 | 205513 | rs2012736 | 0.055378568 | 0.024684132 | 0.024865328 |
| 68 | 205513 | rs2037511 | 0.05703218 | 0.024619463 | 0.020528338 |
| 69 | 205513 | rs2060793 | 0.051548165 | 0.025518202 | 0.043377708 |
| 70 | 205513 | rs2074735 | 0.055322687 | 0.024535941 | 0.024148325 |
| 71 | 205513 | rs212100 | 0.063732039 | 0.024719582 | 0.009931635 |
| 72 | 205513 | rs2123930 | 0.054555204 | 0.024310295 | 0.024824723 |
| 73 | 205513 | rs2131925 | 0.057779864 | 0.024644752 | 0.019052107 |
| 74 | 205513 | rs2207132 | 0.056583336 | 0.024623892 | 0.021567264 |
| 75 | 205513 | rs2229742 | 0.055598655 | 0.024585538 | 0.023732188 |
| 76 | 205513 | rs2248551 | 0.058052616 | 0.024565446 | 0.018118775 |
| 77 | 205513 | rs2278892 | 0.055476371 | 0.02461306 | 0.024199915 |
| 78 | 205513 | rs2346264 | 0.056561109 | 0.024622501 | 0.021611298 |
| 79 | 205513 | rs2352974 | 0.055220112 | 0.024579746 | 0.02466754 |
| 80 | 205513 | rs2528378 | 0.057442735 | 0.02457943 | 0.019437898 |
| 81 | 205513 | rs2535627 | 0.054838428 | 0.024443557 | 0.024866417 |
| 82 | 205513 | rs2585442 | 0.056189214 | 0.024741749 | 0.023145258 |
| 83 | 205513 | rs261291 | 0.056805415 | 0.024702192 | 0.021470309 |
| 84 | 205513 | rs2659007 | 0.05715737 | 0.02460224 | 0.020165256 |
| 85 | 205513 | rs2710647 | 0.058354585 | 0.024430671 | 0.016913678 |
| 86 | 205513 | rs2756119 | 0.055733971 | 0.024561667 | 0.023259543 |
| 87 | 205513 | rs2762943 | 0.05679336 | 0.024698545 | 0.021478731 |
| 88 | 205513 | rs2847500 | 0.056464219 | 0.024631958 | 0.021887442 |
| 89 | 205513 | rs28855697 | 0.056946941 | 0.024620111 | 0.020721244 |
| 90 | 205513 | rs293435 | 0.054951533 | 0.024536108 | 0.025115668 |
| 91 | 205513 | rs2952289 | 0.054633089 | 0.024396509 | 0.025131108 |
| 92 | 205513 | rs34284484 | 0.057345179 | 0.024589551 | 0.019695675 |
| 93 | 205513 | rs34726834 | 0.056626205 | 0.024625125 | 0.021474883 |
| 94 | 205513 | rs34760417 | 0.059300264 | 0.024720896 | 0.0164493 |
| 95 | 205513 | rs35285316 | 0.056801556 | 0.024618821 | 0.02104138 |
| 96 | 205513 | rs35408430 | 0.057537719 | 0.024650861 | 0.019590178 |
| 97 | 205513 | rs35656734 | 0.055658415 | 0.024555982 | 0.023415104 |
| 98 | 205513 | rs3787557 | 0.056655211 | 0.02462827 | 0.021424793 |
| 99 | 205513 | rs3814995 | 0.056312965 | 0.024613503 | 0.022143984 |
| 100 | 205513 | rs41563 | 0.056406373 | 0.024615994 | 0.021937274 |
| 101 | 205513 | rs4364259 | 0.056331749 | 0.024625909 | 0.0221667 |
| 102 | 205513 | rs4418728 | 0.056813979 | 0.024619673 | 0.021017728 |
| 103 | 205513 | rs4565433 | 0.056311661 | 0.024609878 | 0.022127444 |
| 104 | 205513 | rs4575545 | 0.056030788 | 0.02460918 | 0.022796701 |
| 105 | 205513 | rs4616820 | 0.054805157 | 0.02442035 | 0.024816885 |
| 106 | 205513 | rs512083 | 0.057793011 | 0.024549539 | 0.018565701 |
| 107 | 205513 | rs532436 | 0.056251159 | 0.024613437 | 0.022290309 |
| 108 | 205513 | rs541041 | 0.056945836 | 0.024619176 | 0.020718883 |
| 109 | 205513 | rs55683806 | 0.056894011 | 0.024619774 | 0.020837986 |
| 110 | 205513 | rs55707527 | 0.053666925 | 0.024112498 | 0.026035011 |
| 111 | 205513 | rs55814693 | 0.0554736 | 0.024507241 | 0.023601216 |
| 112 | 205513 | rs55829990 | 0.056146304 | 0.024648531 | 0.022733948 |
| 113 | 205513 | rs55872725 | 0.052566699 | 0.023318122 | 0.024175532 |
| 114 | 205513 | rs56019902 | 0.05600949 | 0.024631803 | 0.022973872 |
| 115 | 205513 | rs57459725 | 0.056945609 | 0.024614888 | 0.020697248 |
| 116 | 205513 | rs5770982 | 0.056941219 | 0.024614138 | 0.020703174 |
| 117 | 205513 | rs58038553 | 0.057018539 | 0.024621752 | 0.02057033 |
| 118 | 205513 | rs58387006 | 0.057998816 | 0.024498922 | 0.017913424 |
| 119 | 205513 | rs58411334 | 0.056748039 | 0.024633427 | 0.021239458 |
| 120 | 205513 | rs6011153 | 0.055440047 | 0.024509094 | 0.023696203 |
| 121 | 205513 | rs6123359 | 0.057214379 | 0.024657265 | 0.020319991 |
| 122 | 205513 | rs61887421 | 0.05768207 | 0.024551629 | 0.018802719 |
| 123 | 205513 | rs61891388 | 0.05642614 | 0.024617553 | 0.021899299 |
| 124 | 205513 | rs6438900 | 0.057244623 | 0.024599802 | 0.019963354 |
| 125 | 205513 | rs6671730 | 0.056663839 | 0.024627236 | 0.021399505 |
| 126 | 205513 | rs6672758 | 0.054878758 | 0.024414515 | 0.024589438 |
| 127 | 205513 | rs6724965 | 0.056615773 | 0.024626352 | 0.021505423 |
| 128 | 205513 | rs6782190 | 0.05535955 | 0.024608343 | 0.024472743 |
| 129 | 205513 | rs6837680 | 0.063894541 | 0.025394341 | 0.011866363 |
| 130 | 205513 | rs6857 | 0.05726585 | 0.024630725 | 0.020073233 |
| 131 | 205513 | rs71467497 | 0.056765563 | 0.024622354 | 0.021141483 |
| 132 | 205513 | rs7244811 | 0.056805791 | 0.02462474 | 0.021062708 |
| 133 | 205513 | rs7248342 | 0.058684976 | 0.024571666 | 0.016925645 |
| 134 | 205513 | rs72834856 | 0.057773886 | 0.024566137 | 0.018684361 |
| 135 | 205513 | rs72862131 | 0.057077157 | 0.024620535 | 0.020434411 |
| 136 | 205513 | rs72862854 | 0.052141128 | 0.02432413 | 0.032065203 |
| 137 | 205513 | rs73413596 | 0.054958706 | 0.02442482 | 0.024441507 |
| 138 | 205513 | rs7367758 | 0.053954187 | 0.024195729 | 0.025753726 |
| 139 | 205513 | rs736894 | 0.061936964 | 0.025589303 | 0.015502419 |
| 140 | 205513 | rs7412 | 0.057073635 | 0.024648674 | 0.020586407 |
| 141 | 205513 | rs7439366 | 0.056438632 | 0.024747505 | 0.022573394 |
| 142 | 205513 | rs7528419 | 0.053042078 | 0.024217478 | 0.028506869 |
| 143 | 205513 | rs75419061 | 0.056165248 | 0.024606238 | 0.022456218 |
| 144 | 205513 | rs75604577 | 0.057012723 | 0.02461791 | 0.020563498 |
| 145 | 205513 | rs7569755 | 0.056703261 | 0.024626106 | 0.021303212 |
| 146 | 205513 | rs75865451 | 0.057199517 | 0.024607833 | 0.020101737 |
| 147 | 205513 | rs7604788 | 0.057121981 | 0.024617198 | 0.020318735 |
| 148 | 205513 | rs77037130 | 0.056885028 | 0.024635921 | 0.020942037 |
| 149 | 205513 | rs7784802 | 0.056745203 | 0.024626255 | 0.021208259 |
| 150 | 205513 | rs77960347 | 0.056529342 | 0.024621351 | 0.021678853 |
| 151 | 205513 | rs78168201 | 0.056879425 | 0.024682587 | 0.021198218 |
| 152 | 205513 | rs78649910 | 0.055401661 | 0.024531541 | 0.023921673 |
| 153 | 205513 | rs78886843 | 0.057462359 | 0.024588297 | 0.019440222 |
| 154 | 205513 | rs8018720 | 0.057406599 | 0.024679031 | 0.020011692 |
| 155 | 205513 | rs804281 | 0.055669197 | 0.024607066 | 0.023677287 |
| 156 | 205513 | rs8091117 | 0.057189814 | 0.024607684 | 0.020122106 |
| 157 | 205513 | rs8107974 | 0.060571574 | 0.024278541 | 0.012600664 |
| 158 | 205513 | rs8114057 | 0.056673205 | 0.024620679 | 0.021343415 |
| 159 | 205513 | rs867772 | 0.055414992 | 0.024556525 | 0.024031203 |
| 160 | 205513 | rs9325107 | 0.057016464 | 0.024612932 | 0.020529641 |
| 161 | 205513 | rs9409266 | 0.055054821 | 0.024465161 | 0.024427651 |
| 162 | 205513 | rs9476310 | 0.055678611 | 0.024543249 | 0.023293164 |
| 163 | 205513 | rs949177 | 0.058555487 | 0.024813975 | 0.018285846 |
| 164 | 205513 | rs964184 | 0.059056671 | 0.024670976 | 0.01667615 |
| 165 | 205513 | rs9735104 | 0.05938597 | 0.024961704 | 0.017355757 |
| 166 | 205513 | rs9861009 | 0.056557095 | 0.024625436 | 0.021636224 |
| 167 | 205513 | All | 0.056682163 | 0.024525325 | 0.020823541 |

**Abbreviations:** SE, standard error; SNP, single nucleotide polymorphism.
