# Supplementary material for: A Causal Effect of Serum 25(OH)D Level on Appendicular Muscle Mass: Evidence From NHANES Data and Mendelian Randomization Analyses
Source: J Cachexia Sarcopenia Muscle. 2025 Mar 31;16(2):e13778. doi: 10.1002/jcsm.13778 (PMC11955837; doi:10.1002/jcsm.13778)
Supplement: Supplementary file 6 — Table S6. The pleiotropy effects for the genetic instrumental variables in the two‐sample Mendelian randomization studies to evaluate whether the causality estimate in females was affected by a single SNP using leave‐one‐out methods based on conventional inverse variance weighted model. [file JCSM-16-e13778-s004.docx]

**Supplementary Table 6**. The pleiotropy effects for the genetic instrumental variables in the two-sample Mendelian randomization studies to evaluate whether the causality estimate in females was affected by a single SNP using leave-one-out methods based on conventional inverse variance weighted model.

| Female | Sample size | SNP | *β* | *SE* | *P* |
| --- | --- | --- | --- | --- | --- |
| 1 | 244730 | rs10008500 | 0.041901132 | 0.025172217 | 0.095996869 |
| 2 | 244730 | rs10083762 | 0.04229157 | 0.025159033 | 0.09276882 |
| 3 | 244730 | rs1038165 | 0.041642929 | 0.025109275 | 0.097223045 |
| 4 | 244730 | rs1047891 | 0.046759697 | 0.023931271 | 0.050711137 |
| 5 | 244730 | rs10832164 | 0.042857423 | 0.025218999 | 0.089241929 |
| 6 | 244730 | rs10859995 | 0.043185688 | 0.02541983 | 0.089338487 |
| 7 | 244730 | rs10880925 | 0.042108804 | 0.025158303 | 0.094179003 |
| 8 | 244730 | rs10887718 | 0.042799941 | 0.025162526 | 0.088954295 |
| 9 | 244730 | rs10896045 | 0.04379094 | 0.025056517 | 0.080518306 |
| 10 | 244730 | rs10908465 | 0.043761291 | 0.025112962 | 0.081407387 |
| 11 | 244730 | rs11076175 | 0.040443868 | 0.025064682 | 0.106618571 |
| 12 | 244730 | rs11127186 | 0.04288723 | 0.025157177 | 0.088237114 |
| 13 | 244730 | rs11204743 | 0.044517785 | 0.025002313 | 0.074986546 |
| 14 | 244730 | rs11249443 | 0.042758675 | 0.025162272 | 0.08925949 |
| 15 | 244730 | rs11264223 | 0.040653063 | 0.024927019 | 0.102914919 |
| 16 | 244730 | rs113256381 | 0.043274656 | 0.025132629 | 0.08509641 |
| 17 | 244730 | rs113292111 | 0.044339854 | 0.025161165 | 0.078029805 |
| 18 | 244730 | rs114204813 | 0.041978992 | 0.025242839 | 0.096311237 |
| 19 | 244730 | rs114687675 | 0.042812453 | 0.025200808 | 0.089347007 |
| 20 | 244730 | rs1149610 | 0.043778418 | 0.02512827 | 0.081473832 |
| 21 | 244730 | rs11542462 | 0.043334703 | 0.02516779 | 0.085099976 |
| 22 | 244730 | rs11591147 | 0.043239698 | 0.025138555 | 0.085422446 |
| 23 | 244730 | rs116778432 | 0.042614115 | 0.025172675 | 0.090479849 |
| 24 | 244730 | rs117287238 | 0.043256713 | 0.025164715 | 0.085624728 |
| 25 | 244730 | rs11732896 | 0.041236425 | 0.025097011 | 0.100366147 |
| 26 | 244730 | rs117363662 | 0.043307072 | 0.025268511 | 0.086551669 |
| 27 | 244730 | rs118055554 | 0.042351572 | 0.025163914 | 0.09236967 |
| 28 | 244730 | rs11826004 | 0.041403923 | 0.025173389 | 0.100021446 |
| 29 | 244730 | rs12056768 | 0.041255646 | 0.025190038 | 0.101468384 |
| 30 | 244730 | rs12123821 | 0.046751481 | 0.025195036 | 0.06351302 |
| 31 | 244730 | rs1229984 | 0.042116176 | 0.0251627 | 0.09417891 |
| 32 | 244730 | rs12307364 | 0.042861344 | 0.025184926 | 0.088780681 |
| 33 | 244730 | rs12317268 | 0.04171952 | 0.025145895 | 0.097095915 |
| 34 | 244730 | rs12462826 | 0.043059126 | 0.025153891 | 0.086928435 |
| 35 | 244730 | rs1247583 | 0.043023343 | 0.02515671 | 0.087226412 |
| 36 | 244730 | rs12501515 | 0.043182455 | 0.026034997 | 0.097190159 |
| 37 | 244730 | rs12507691 | 0.041803144 | 0.025125929 | 0.096163357 |
| 38 | 244730 | rs1260326 | 0.033564473 | 0.0234396 | 0.152156442 |
| 39 | 244730 | rs12775091 | 0.042166898 | 0.025159034 | 0.093735418 |
| 40 | 244730 | rs12949853 | 0.040237112 | 0.02477513 | 0.10435599 |
| 41 | 244730 | rs13284054 | 0.042222592 | 0.025159671 | 0.093310914 |
| 42 | 244730 | rs139415780 | 0.042366113 | 0.02518633 | 0.092548 |
| 43 | 244730 | rs139959724 | 0.040805375 | 0.025164531 | 0.104901182 |
| 44 | 244730 | rs140371183 | 0.044111552 | 0.025274961 | 0.080938422 |
| 45 | 244730 | rs140433285 | 0.041960793 | 0.02515255 | 0.095265697 |
| 46 | 244730 | rs142004400 | 0.043777072 | 0.025065774 | 0.080726202 |
| 47 | 244730 | rs142158911 | 0.041695016 | 0.025173535 | 0.097660349 |
| 48 | 244730 | rs142369684 | 0.044458222 | 0.025154612 | 0.077161522 |
| 49 | 244730 | rs143069752 | 0.041651624 | 0.025106675 | 0.097118601 |
| 50 | 244730 | rs143488652 | 0.043268363 | 0.025141384 | 0.085250525 |
| 51 | 244730 | rs143645388 | 0.042376882 | 0.025167622 | 0.092223095 |
| 52 | 244730 | rs148843488 | 0.043502746 | 0.025141648 | 0.083575606 |
| 53 | 244730 | rs150270324 | 0.044041802 | 0.025102491 | 0.079348398 |
| 54 | 244730 | rs16846771 | 0.041538104 | 0.025300152 | 0.100628881 |
| 55 | 244730 | rs17144574 | 0.041613123 | 0.025129793 | 0.097736436 |
| 56 | 244730 | rs1792329 | 0.044058305 | 0.02519083 | 0.080294153 |
| 57 | 244730 | rs1792556 | 0.043157474 | 0.025160006 | 0.086286481 |
| 58 | 244730 | rs1800588 | 0.043483597 | 0.025243405 | 0.084965845 |
| 59 | 244730 | rs182244780 | 0.049880041 | 0.026085433 | 0.055853119 |
| 60 | 244730 | rs183409297 | 0.042669291 | 0.02517417 | 0.090082402 |
| 61 | 244730 | rs1858889 | 0.043067469 | 0.025147921 | 0.086792462 |
| 62 | 244730 | rs1872285 | 0.044148752 | 0.02504673 | 0.077958632 |
| 63 | 244730 | rs187706948 | 0.043330667 | 0.025154838 | 0.084968492 |
| 64 | 244730 | rs188247550 | 0.042319207 | 0.025169157 | 0.092686891 |
| 65 | 244730 | rs189407772 | 0.042882585 | 0.025177536 | 0.088529123 |
| 66 | 244730 | rs1966478 | 0.041828672 | 0.025128618 | 0.095995972 |
| 67 | 244730 | rs2012736 | 0.042894337 | 0.025252402 | 0.089390046 |
| 68 | 244730 | rs2037511 | 0.043350684 | 0.025134579 | 0.084573836 |
| 69 | 244730 | rs2060793 | 0.041934068 | 0.026129758 | 0.108529125 |
| 70 | 244730 | rs2074735 | 0.040404691 | 0.024948931 | 0.105340619 |
| 71 | 244730 | rs212100 | 0.037371665 | 0.025375048 | 0.14081247 |
| 72 | 244730 | rs2123930 | 0.041673188 | 0.025115028 | 0.097056785 |
| 73 | 244730 | rs2131925 | 0.043867377 | 0.025179062 | 0.081470563 |
| 74 | 244730 | rs2207132 | 0.041816434 | 0.025137382 | 0.096209222 |
| 75 | 244730 | rs2229742 | 0.04291316 | 0.025174963 | 0.088269661 |
| 76 | 244730 | rs2248551 | 0.042834959 | 0.025180674 | 0.088923357 |
| 77 | 244730 | rs2278892 | 0.041404412 | 0.025162252 | 0.099867346 |
| 78 | 244730 | rs2346264 | 0.043048498 | 0.025152012 | 0.086982766 |
| 79 | 244730 | rs2352974 | 0.04293698 | 0.025188327 | 0.088261946 |
| 80 | 244730 | rs2528378 | 0.042445204 | 0.025164876 | 0.091664047 |
| 81 | 244730 | rs2535627 | 0.041570512 | 0.025121705 | 0.097972228 |
| 82 | 244730 | rs2585442 | 0.043728549 | 0.025276045 | 0.083623271 |
| 83 | 244730 | rs261291 | 0.042602307 | 0.025247689 | 0.091531359 |
| 84 | 244730 | rs2659007 | 0.042336244 | 0.025160684 | 0.092445798 |
| 85 | 244730 | rs2710647 | 0.042512895 | 0.025166677 | 0.091170879 |
| 86 | 244730 | rs2756119 | 0.041120836 | 0.025030537 | 0.100418785 |
| 87 | 244730 | rs2762943 | 0.04165994 | 0.025231573 | 0.098717432 |
| 88 | 244730 | rs2847500 | 0.042531359 | 0.025178919 | 0.091187733 |
| 89 | 244730 | rs28855697 | 0.043385205 | 0.025126212 | 0.084223166 |
| 90 | 244730 | rs293435 | 0.041211094 | 0.025125349 | 0.100959476 |
| 91 | 244730 | rs2952289 | 0.041249681 | 0.025081106 | 0.100041681 |
| 92 | 244730 | rs34284484 | 0.042490677 | 0.025165221 | 0.091321371 |
| 93 | 244730 | rs34726834 | 0.042701799 | 0.025168365 | 0.089763662 |
| 94 | 244730 | rs34760417 | 0.043608864 | 0.025317741 | 0.084985732 |
| 95 | 244730 | rs35285316 | 0.042455127 | 0.025163605 | 0.09157183 |
| 96 | 244730 | rs35408430 | 0.044920122 | 0.025087743 | 0.073370251 |
| 97 | 244730 | rs35656734 | 0.041399661 | 0.025084766 | 0.098863076 |
| 98 | 244730 | rs3787557 | 0.042169309 | 0.025165082 | 0.093795553 |
| 99 | 244730 | rs3814995 | 0.042237686 | 0.025160708 | 0.093207383 |
| 100 | 244730 | rs41563 | 0.041979504 | 0.025143136 | 0.094994401 |
| 101 | 244730 | rs4364259 | 0.042435517 | 0.025175628 | 0.091876864 |
| 102 | 244730 | rs4418728 | 0.043173816 | 0.025137543 | 0.085887233 |
| 103 | 244730 | rs4565433 | 0.042753109 | 0.025161619 | 0.089292848 |
| 104 | 244730 | rs4575545 | 0.040751567 | 0.025014461 | 0.103287546 |
| 105 | 244730 | rs4616820 | 0.04045182 | 0.024916721 | 0.104486653 |
| 106 | 244730 | rs512083 | 0.044134626 | 0.025019219 | 0.077727115 |
| 107 | 244730 | rs532436 | 0.042715312 | 0.02516756 | 0.089651872 |
| 108 | 244730 | rs541041 | 0.042881137 | 0.025161194 | 0.088333118 |
| 109 | 244730 | rs55683806 | 0.042635002 | 0.025166688 | 0.090245405 |
| 110 | 244730 | rs55707527 | 0.039164371 | 0.024534795 | 0.110426579 |
| 111 | 244730 | rs55814693 | 0.041250293 | 0.025036473 | 0.099433143 |
| 112 | 244730 | rs55829990 | 0.044623907 | 0.025085905 | 0.07526537 |
| 113 | 244730 | rs55872725 | 0.040098855 | 0.024716569 | 0.104729034 |
| 114 | 244730 | rs56019902 | 0.042802434 | 0.025189478 | 0.08927797 |
| 115 | 244730 | rs57459725 | 0.042480715 | 0.025164114 | 0.091383077 |
| 116 | 244730 | rs5770982 | 0.043198247 | 0.02513153 | 0.085635041 |
| 117 | 244730 | rs58038553 | 0.043823984 | 0.025085602 | 0.080641234 |
| 118 | 244730 | rs58387006 | 0.043997929 | 0.025020435 | 0.078665903 |
| 119 | 244730 | rs58411334 | 0.043022435 | 0.025168563 | 0.087382077 |
| 120 | 244730 | rs6011153 | 0.041741804 | 0.025118322 | 0.096551475 |
| 121 | 244730 | rs6123359 | 0.042998421 | 0.025203988 | 0.088004987 |
| 122 | 244730 | rs61887421 | 0.042583038 | 0.025165918 | 0.090628511 |
| 123 | 244730 | rs61891388 | 0.041930857 | 0.025140602 | 0.095344693 |
| 124 | 244730 | rs6438900 | 0.042978661 | 0.025153809 | 0.087518701 |
| 125 | 244730 | rs6671730 | 0.04268344 | 0.025170775 | 0.089932493 |
| 126 | 244730 | rs6672758 | 0.041607752 | 0.025111755 | 0.097539016 |
| 127 | 244730 | rs6724965 | 0.043108117 | 0.02515372 | 0.086567855 |
| 128 | 244730 | rs6782190 | 0.03863456 | 0.024787478 | 0.119083473 |
| 129 | 244730 | rs6837680 | 0.046955283 | 0.026013598 | 0.071070241 |
| 130 | 244730 | rs6857 | 0.043564173 | 0.025151672 | 0.083263107 |
| 131 | 244730 | rs71467497 | 0.043363834 | 0.025123871 | 0.084347182 |
| 132 | 244730 | rs7244811 | 0.043493586 | 0.025118084 | 0.083351101 |
| 133 | 244730 | rs7248342 | 0.042345533 | 0.025209913 | 0.09301229 |
| 134 | 244730 | rs72834856 | 0.044395437 | 0.024997416 | 0.075732744 |
| 135 | 244730 | rs72862131 | 0.043338189 | 0.025141513 | 0.084749345 |
| 136 | 244730 | rs72862854 | 0.037698646 | 0.024826938 | 0.128899147 |
| 137 | 244730 | rs73413596 | 0.041310273 | 0.025066739 | 0.099350319 |
| 138 | 244730 | rs7367758 | 0.040711684 | 0.024980296 | 0.103153944 |
| 139 | 244730 | rs736894 | 0.050980702 | 0.026087988 | 0.050679583 |
| 140 | 244730 | rs7412 | 0.042698452 | 0.025197287 | 0.09015789 |
| 141 | 244730 | rs7439366 | 0.042795512 | 0.025293865 | 0.090658852 |
| 142 | 244730 | rs7528419 | 0.039382653 | 0.024872807 | 0.113339031 |
| 143 | 244730 | rs75419061 | 0.042042724 | 0.025152028 | 0.094614248 |
| 144 | 244730 | rs75604577 | 0.04343436 | 0.025120421 | 0.08380088 |
| 145 | 244730 | rs7569755 | 0.043878912 | 0.02506996 | 0.080073706 |
| 146 | 244730 | rs75865451 | 0.044279873 | 0.024985322 | 0.076355485 |
| 147 | 244730 | rs7604788 | 0.043331219 | 0.025139328 | 0.084772371 |
| 148 | 244730 | rs77037130 | 0.04265523 | 0.025182067 | 0.09028937 |
| 149 | 244730 | rs7784802 | 0.041363382 | 0.025091539 | 0.099250277 |
| 150 | 244730 | rs77960347 | 0.041915028 | 0.025139715 | 0.095458075 |
| 151 | 244730 | rs78168201 | 0.042591272 | 0.025232024 | 0.091414157 |
| 152 | 244730 | rs78649910 | 0.040259118 | 0.02486639 | 0.105443511 |
| 153 | 244730 | rs78886843 | 0.042653698 | 0.025169248 | 0.090137068 |
| 154 | 244730 | rs8018720 | 0.041005511 | 0.025194407 | 0.103617359 |
| 155 | 244730 | rs804281 | 0.041213298 | 0.025126099 | 0.100951431 |
| 156 | 244730 | rs8091117 | 0.042467714 | 0.025168224 | 0.091535224 |
| 157 | 244730 | rs8107974 | 0.04613697 | 0.02488615 | 0.063750386 |
| 158 | 244730 | rs8114057 | 0.042922866 | 0.025154952 | 0.087945057 |
| 159 | 244730 | rs867772 | 0.040967132 | 0.025060386 | 0.102104266 |
| 160 | 244730 | rs9325107 | 0.041809073 | 0.025126492 | 0.096123648 |
| 161 | 244730 | rs9409266 | 0.041582356 | 0.025114119 | 0.097775247 |
| 162 | 244730 | rs9476310 | 0.042421337 | 0.02516331 | 0.091826448 |
| 163 | 244730 | rs949177 | 0.042371606 | 0.025389331 | 0.095142265 |
| 164 | 244730 | rs964184 | 0.041469112 | 0.025269572 | 0.100783087 |
| 165 | 244730 | rs9735104 | 0.043356883 | 0.025537961 | 0.089556362 |
| 166 | 244730 | rs9861009 | 0.042837753 | 0.025166165 | 0.088718571 |
| 167 | 244730 | All | 0.042549691 | 0.025067684 | 0.089622535 |

**Abbreviations:** SE, standard error; SNP, single nucleotide polymorphism.
